# On-Surface Synthesis of a Ferromagnetic Molecular Spin Trimer

**DOI:** 10.1021/jacs.4c15736

**Published:** 2025-05-30

**Authors:** Alessio Vegliante, Manuel Vilas-Varela, Ricardo Ortiz, Francisco Romero Lara, Manish Kumar, Lucía Gómez-Rodrigo, Stefano Trivini, Fabian Schulz, Diego Soler-Polo, Hassan Ahmoum, Emilio Artacho, Thomas Frederiksen, Pavel Jelínek, Jose Ignacio Pascual, Diego Peña

**Affiliations:** † 138823 CIC NanoGUNE-BRTA, Donostia-San Sebastián 20018, Spain; ‡ Centro Singular de Investigación en Química Biolóxica e Materiais Moleculares (CiQUS) and Departamento de Química Orgánica, 16780 Universidade de Santiago de Compostela, Santiago de Compostela 15782, Spain; § Donostia International Physics Center (DIPC), Donostia-San Sebastián 20018, Spain; ∥ Institute of Physics, Czech Academy of Sciences, Prague 16200, Czech Republic; ⊥ Materials Physics Center (CFM-MPC), Donostia-San Sebastián E-20018, Spain; # Ikerbasque, Basque Foundation for Science, Bilbao 48013, Spain; ¶ Theory of Condensed Matter, Cavendish Laboratory, University of Cambridge, Cambridge CB3 0HE, U.K.; ∇ Czech Advanced Technology and Research Institute (CATRIN), Palacký University Olomouc, Olomouc 77900, Czech Republic; ○ Oportunius, Galician Innovation Agency (GAIN), Santiago de Compostela 15702, Spain

## Abstract

Triangulenes are prototypical examples of open-shell nanographenes. Their magnetic properties, arising from the presence of unpaired π electrons, can be extensively tuned by modifying their size and shape or by introducing heteroatoms. Different triangulene derivatives have been designed and synthesized in recent years thanks to the development of on-surface synthesis strategies. Triangulene-based nanostructures with polyradical character, hosting several interacting spin units, can be challenging to fabricate but are particularly interesting for potential applications in carbon-based spintronics. Here, we combine pristine and N-doped triangulenes into a more complex nanographene, **TTAT**, predicted to possess three unpaired π electrons delocalized along the zigzag periphery. We generate the molecule on a Au(111) surface and detect direct fingerprints of multiradical coupling and high-spin state using scanning tunneling microscopy and spectroscopy. With the support of theoretical calculations, we show that its three radical units are localized at distinct parts of the molecule and couple via symmetric ferromagnetic interactions, which result in a *S* = 3/2 ground state, thus demonstrating the realization of a molecular ferromagnetic Heisenberg spin trimer.

## Graphical abstract

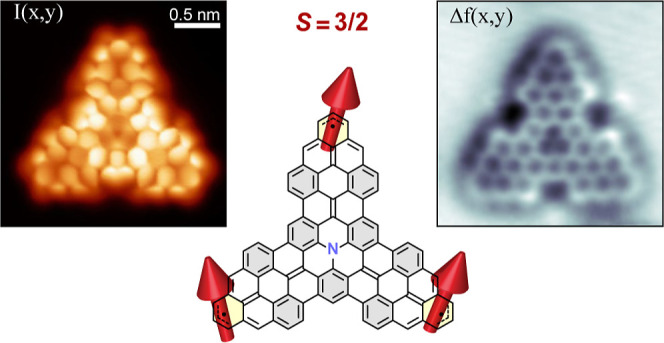

## Introduction

Recent advances in molecular nanoscience have shown that small graphene flakes with atomically customized shapes can exhibit π-paramagnetism associated with radical states in open-shell structures. This unique form of magnetism exhibits distinctive characteristics such as spin delocalization and large spin exchange interactions.^1,2^ Owing to the weak spin–orbit coupling in carbon compounds, spin-hosting nanographenes are anticipated to exhibit long spin-coherence times, making them promising candidates for applications in quantum computing.^3^ The spin ground state of a magnetic nanographene is determined by its atomic-scale structure and composition.^4,5^ Therefore, atomically precise on-surface synthesis (OSS) techniques,^6^ in combination with the solution synthesis of organic precursors, offer a unique opportunity to engineer novel magnetic states in two dimensions through the creation of unpaired electrons at radical sites.

Graphene flakes with triangular shapes are paradigmatic platforms for hosting interacting quantum spins. Ovchinnikov’s rule^7^ for alternant conjugated lattices predicts an intrinsic spin imbalance. The net spin of triangulene molecules, for example, increases with the flake’s size and can also be modified by heteroatom substitution.^8–15^ The high-spin states of triangulenes are exceptionally robust because their singly occupied orbitals (or zero-energy states) live in the same carbon sublattice, thus having a large spatial wave function overlap. Consequently, Hund’s exchange coupling and spin excitation energies generally amount to a significant fraction of an electronvolt. While systems with considerable energy gaps between the ground and excited spin states can be attractive for some applications related to classical magnetism, this can be a drawback for applications utilizing the full spin spectrum of the nanographene.

Nanographenes with weakly interacting spins have been successfully synthesized on surfaces by covalently bonding triangulene building blocks through their vertices.^12,16–19,21^ This strategy maintains the triangulene integrity because the zero-energy modes have a low density of states over these connecting sites. As a result, collective spin states emerge in polyradical chains and rings from antiferromagnetic interactions between the monomer units. However, there is scarce direct evidence of ferromagnetic exchange interactions between weakly coupled radical states that would allow us to investigate the full spin excitation spectrum. An alternative strategy rarely explored is connecting the nanographenes through their zigzag edges. While this method modifies the electronic and magnetic configuration of the original structure,^22^ it can build customized flakes with interacting localized radicals.^23^

In this study, we implemented this connection strategy to synthesize a large triangular nanographene with a symmetric trimer of ferromagnetically interacting localized radicals. By fusing three [3]­triangulenes (3T) onto the edges of an aza[3]­triangulene (A3T) core, we formed TTAT (tris-triangulene-aza-triangulene), a high-spin triradical nanographene. Each 3T unit hosts two unpaired π electrons localized along majority zigzag sites.^8,9^ As shown in Figure 1a, this connection strategy pairs majority sublattice sites in opposite orientations, reducing the number of zero-energy states (i.e., their nullity^24,25^ ) from eight to four and preserving four unpaired π electrons for the pristine carbon structure.

**Figure 1:**
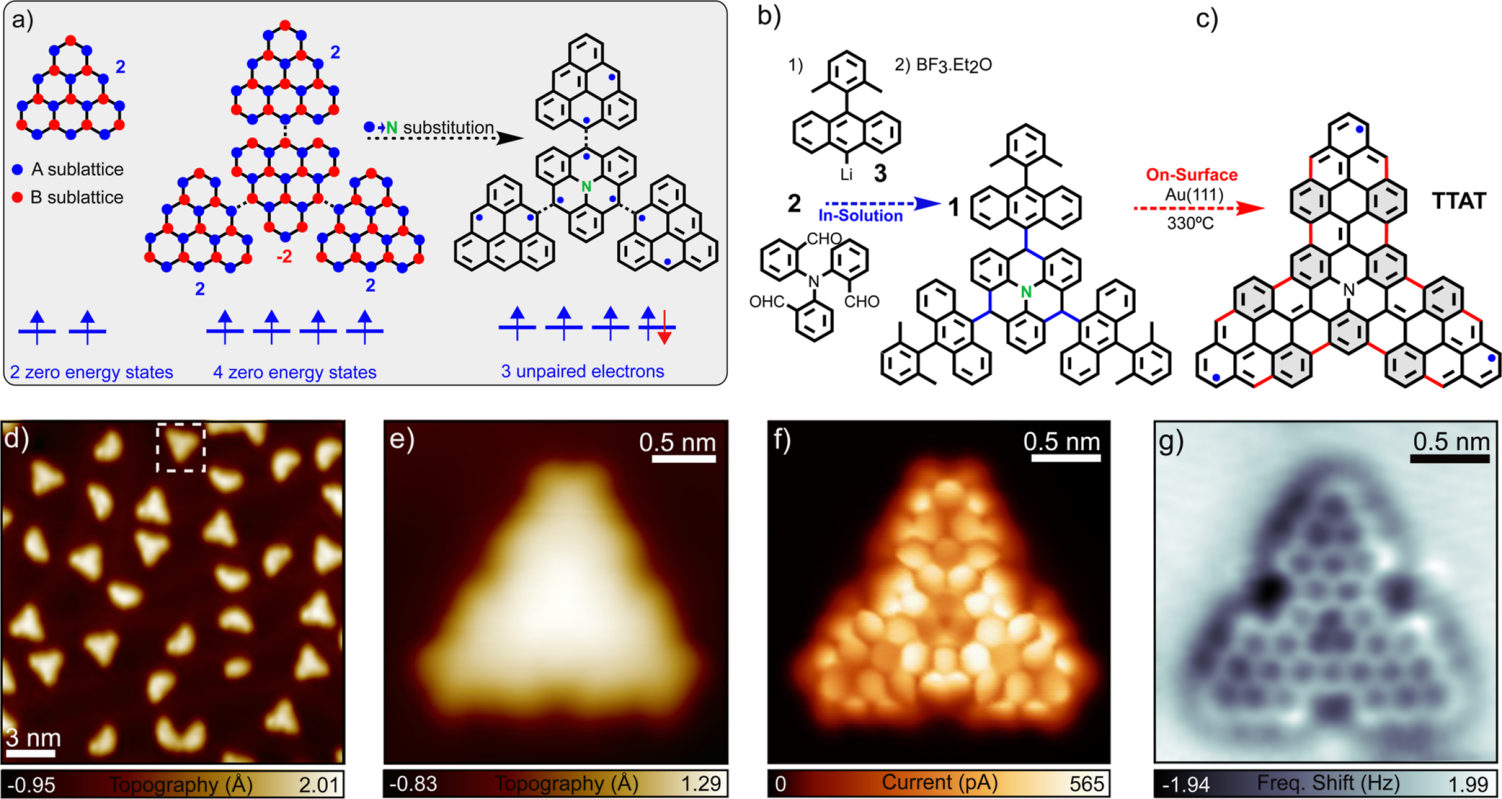
(a) Schematic representation of the formation of the aza-triradical (**TTAT**) in (c) from four [3]­triangulenes combined such that the majority sublattice of the central triangulene results opposite to the one of the three external triangulenes. Introducing a N atom in the center of the inner triangulene (in the majority sublattice) results in five electrons occupying four states. The final structure, therefore, hosts three unpaired electrons, expected to couple ferromagnetically by Hund’s exchange. (b) In-solution and on-surface reaction steps leading to the synthesis of **TTAT**, with the C–C bonds formed during each step indicated in blue and red, respectively. (d) STM constant-current image (*V* = 0.9 V, *I* = 30 pA) after deposition of the precursor on Au(111) and subsequent annealing at 330 °C. The white dotted square highlights an intact and planar molecule, corresponding to **TTAT**. (e) STM constant-current image of **TTAT** measured with a CO-functionalized tip (*V* = 200 mV, *I* = 30 pA). (f) Constant-height BR STM current scan (*V* = 5 mV) and (g) constant-height BR AFM image (oscillation amplitude *A* = 60 pm), performed with CO-functionalized tips.

Within the A3T core, nitrogen substitution at a majority site introduces an additional electron into the π system, stabilizing a *D*_3*h*_ symmetric configuration with three unpaired electrons at the zigzag corners. Structurally, TTAT resembles an aza[8]­triangulene with six fewer six-membered rings, two per triangular side. This change creates three distinct gulf regions along the edges, each formed by two conjoined bay areas that accommodate nine Clar sextets (Figure 1c).

In the following, we report the on-surface generation of **TTAT** on a Au(111) surface and demonstrate that this molecule behaves as a ferromagnetic Heisenberg spin triangle. Combining low-temperature scanning tunneling microscopy (STM) measurements with theoretical simulations, we resolve its structural integrity on a surface and demonstrate that **TTAT** lies on a neutral charge state, maintaining a spin 3/2 ground state. The resolution of low-energy spectroscopic fingerprints and their simulation through multiconfigurational simulations revealed the presence of a triradical character with a ferromagnetic interaction among its unpaired electrons. This spin triangle represents a unique system for investigating entanglement in a single-molecular architecture.

## Results and Discussion

### Synthesis Strategy of TTAT

We envisioned the synthesis of **TTAT** through a combination of in-solution reaction and OSS, as represented in Figure 1b and c, respectively. First, we addressed the preparation of the TTAT precursor **1** following the synthesis strategy of an aza-[5]-triangulene (A5T) precursor^14^ shown schematically in Figure 1b and described in more detail in Section S1. Specifically, we followed a sequence of in-solution reaction steps based on the treatment of tribenzaldehyde **2** with an excess of organolithium **3**, followed by a BF_3_-promoted 3-fold intramolecular Friedel–Crafts reaction. Following this synthetic protocol, we isolated compound **1** in 38% yield. Notably, this two-step synthetic route, which is based on the use of amine **2** as the starting building block, may be used to obtain other complex N-doped nanographenes with 3-fold symmetry in a very simple manner.

We deposited the **TTAT** precursor **1** onto a Au(111) surface at room temperature via the flash annealing of a silicon wafer loaded with molecular grains. Subsequently, the sample was annealed at 330 °C to activate the dehydrogenation reactions necessary to induce the formation of the 12 C–C bonds as shown in red in Figure 1c and culminate in the molecular planarization. The overview STM constant-current image recorded after annealing (Figure 1d) shows intact triangular-shaped products alongside smaller molecular fragments. A closer inspection reveals that many triangular products retain one or more methyl groups, which appear as protruding rounded lobes in the STM images. Nevertheless, we identified the target product, **TTAT**, in a small fraction (around 5%) of the nonfragmented molecules. The molecular structure appears fully planarized in this case and displays chamfered corners (Figure 1e). To conclusively demonstrate the successful on-surface generation of **TTAT**, we performed bond-resolved (BR) constant-height STM and noncontact AFM imaging using a CO-terminated tip^28^ (Figure 1f,g). The BR images resolve the molecular backbone, revealing the absence of structural defects and the preserved 3-fold symmetry of **TTAT** upon adsorption on the surface. Additionally, the BR STM image, recorded at *V* = 5 mV, displays an apparent increase in the current signal along the zigzag edges near the triangulene corners and in the gulf regions around the center, providing a first indication of an enhanced density of states around the Fermi level, likely due to the presence of radical states.^1^

### Evidence of Open-Shell States on Au(111)

To address the electronic and magnetic properties of **TTAT** on Au(111), we performed differential conductance (d*I*/d*V*) spectroscopy (Figure 2a). First, we explored the low-energy spectral window, where spin fingerprints appear in the density of states. Spectra measured on the corners of the outer triangulenes show low-bias features indicative of an open-shell character: a weak zero-bias peak, characteristic of a Kondo resonance, and bias-symmetric step-like features at *V* ≈ ±15 mV, which are related to inelastic spin excitation processes.^1,2,17,18,20,21,23,29–31^ In contrast, spectra measured on other molecular positions (specifically on the N atom and in the gulf regions along the edges, as shown in Figure S5) do not display low-bias features indicative of spin states.

**Figure 2:**
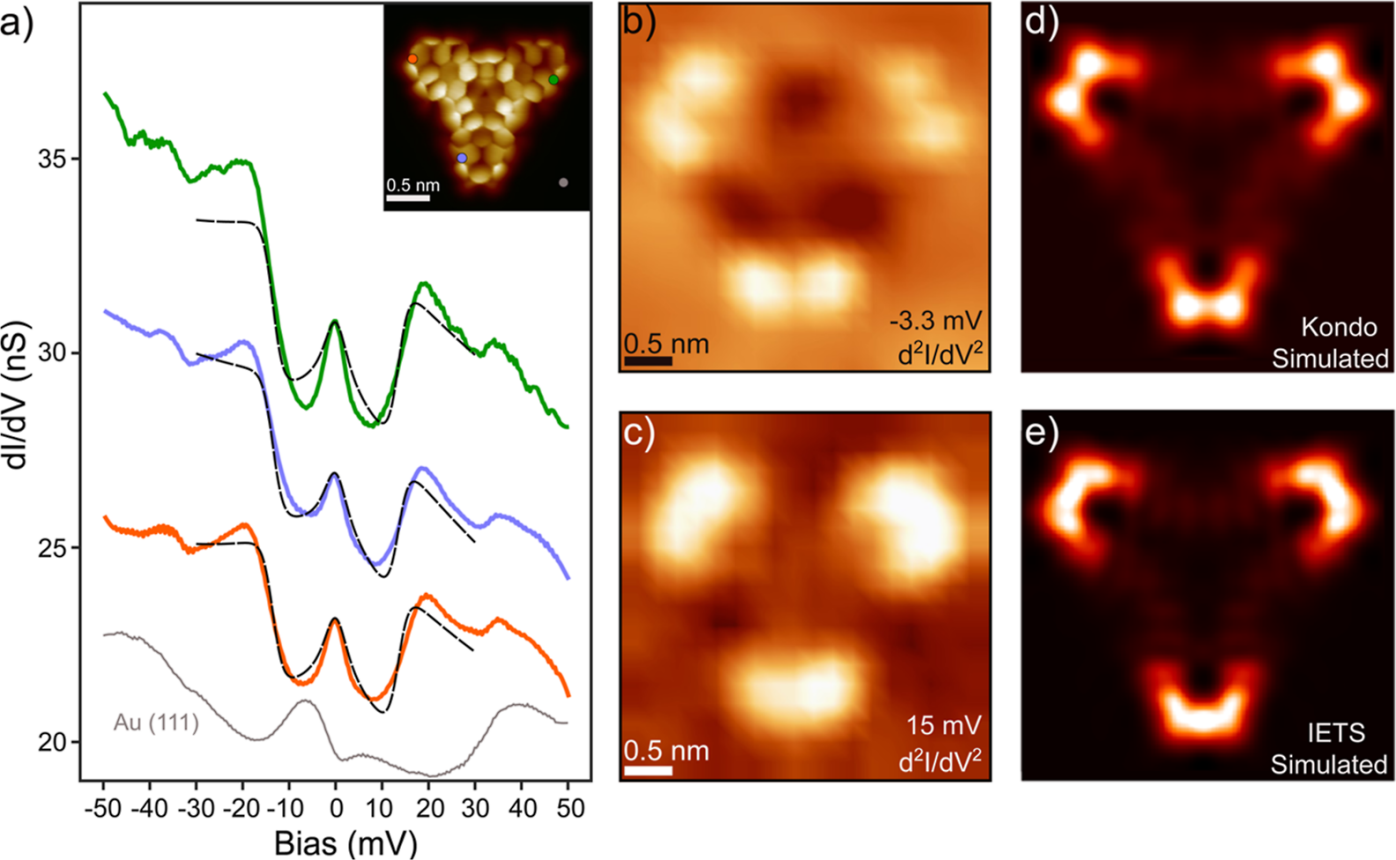
(a) Low-energy d*I*/d*V* spectra of **TTAT** measured with a CO-functionalized tip at the positions indicated in the inset. The spectra display a zero-bias resonance and inelastic spin excitation features at *V* ≈ ±15 mV. The black dashed lines represent fits to the data using the perturbative model by Ternes,^26^ for the case of three *S* = 1/2 spins coupled with a ferromagnetic exchange *J* = 9 meV. Weaker steps at ±35 mV are attributed to the excitation of frustrated rotational modes of the CO molecule.^27^ Spectroscopy parameters: *V* = 50 mV, *I* = 1 nA, and *V*_mod_ = 2 mV. (b,c) d^2^ *I*/d*V* ^2^ maps at *V* = −3.3 mV and *V* = 15 mV, obtained by numerical differentiation from a grid of d*I*/d*V* point spectra (see Methods section for a description of the procedure). The maps probe the spatial distribution of the zero-bias resonance (b) and the inelastic signal (c) without elastic background effects. (d) Simulated Kondo and (e) spin excitation d*I*/d*V* maps, computed from the Kondo orbitals (d) and natural transition orbitals (NTOs), respectively (see text and Supporting Information).

To probe the spatial distribution of both Kondo and inelastic electron tunneling spectroscopy (IETS) features, we mapped the derivative of the differential conductance (i.e., d^2^ *I*/d*V* ^2^ maps) at *V* = −3.27 mV and *V* = 15 mV, respectively. At these bias values, Kondo and IETS features appear as peaks in d^2^ *I*/d*V* ^2^ spectra, with amplitude proportional to the weight of the Kondo and inelastic channels^18^ (Figure S6). As shown in Figure 2b and c, in both maps, the d^2^ *I*/d*V* ^2^ signal appears localized on the corners of the three outer triangulenes, indicating that the radical character of the molecule stems primarily from orbitals distributed over the edge of the external moieties, as we will discuss later.

Resolution of the **TTAT** frontier orbitals and their distribution over the molecular architecture provide a glimpse of the molecular spin ground state. We measured d*I*/d*V* spectra on a broader bias range [1, −1 V] on distinct molecular positions (Figure 3a) and found several peaked resonances attributed to molecular states. The most protruding one is a clear resonance centered at around −100 mV, with spatial distribution over the central aza moiety, as probed by the constant-height d*I*/d*V* map reported in Figure 3b. The tail of this resonance crosses through zero bias and causes the tilted background in the low-energy spectra of Figure 2a. It also accounts for the increased current around the center observed in BR constant-height images like those in Figure 1f. As shown in Figure 3c, a nonvanishing signal over the central aza moiety is only found in the d*I*/d*V* map measured at −100 meV, with no replica at positive bias. This points toward a doubly occupied state over the center of the flake, in agreement with results from DFT calculations reported in Figure S9 for a free molecule. According to DFT, zero-energy orbital A_1_ (with the largest amplitude over the N site and C_3*v*_ symmetry) hosts the extra electron provided by the N heteroatom substitution in the neutral charge state. The molecule spin is primarily hosted in the three remaining singly occupied molecular orbitals (SOMOs), two of which are degenerated, with E symmetry, and the third one is the C_3*v*_ symmetric A_2_ orbital.^32^ These orbitals are distributed mainly along the molecular edges with a weak contribution from the aza group.

**Figure 3:**
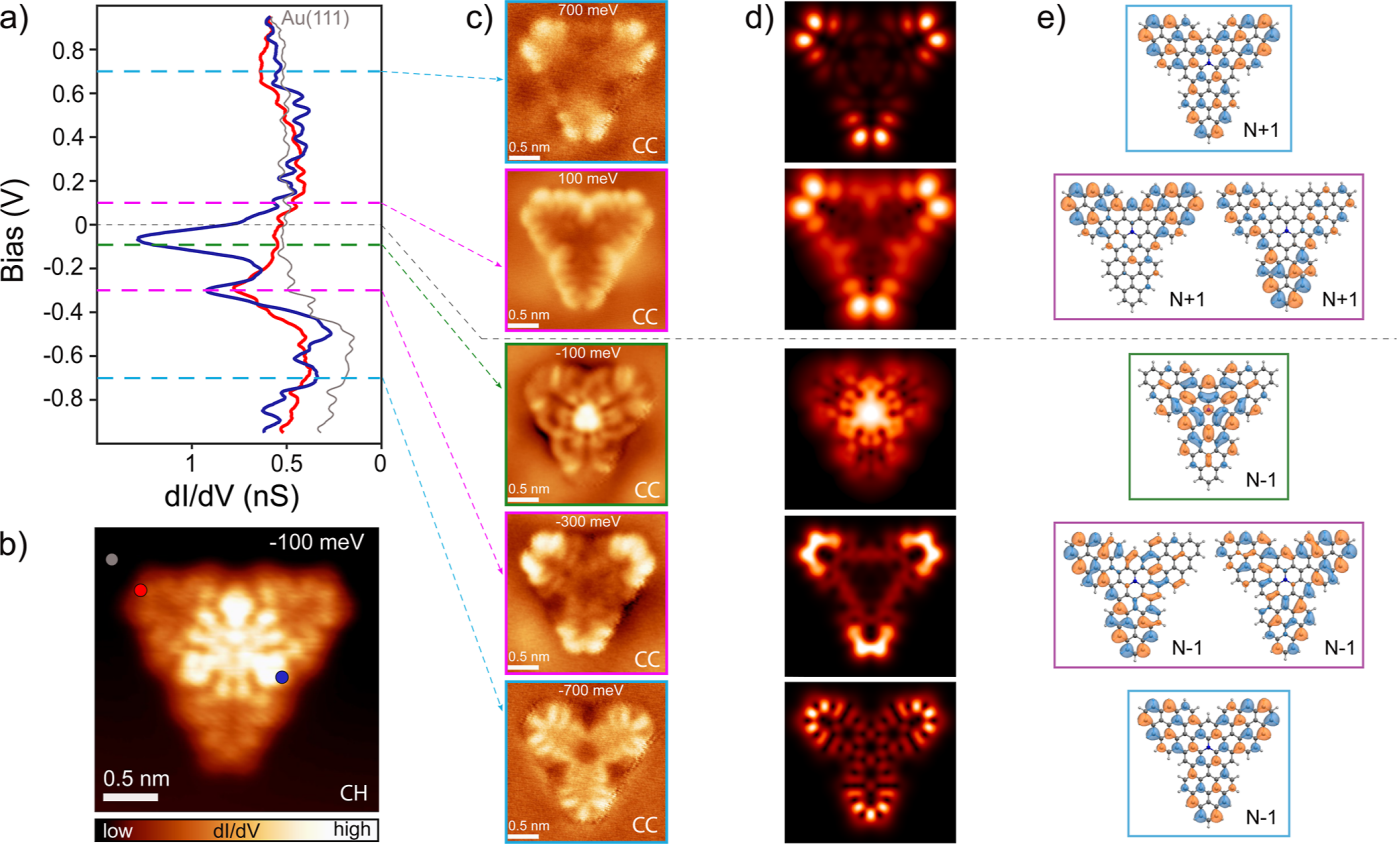
(a) d*I*/d*V* spectra measured at the points indicated in (b), revealing molecular orbital resonances (*V* = 1 V, *I* = 500 pA, and *V*_mod_ = 10 mV). (b) Constant-height (CH) d*I*/d*V* map recorded at *V* = −100 mV with a CO-functionalized tip, corresponding to an orbital with a nonvanishing signal over the inner N-doped triangulene (open feedback parameters: *V* = −100 mV, *I* = 300 pA, and *V*_mod_ = 10 mV). (c) Constant-current (CC) d*I*/d*V* maps recorded at different bias values around 0, with a CO-terminated tip (*I* = 300 pA and *V*_mod_ = 10 mV). (d) Simulated d*I*/d*V* maps, obtained using Dyson orbitals, corresponding to the processes of adding and removing electrons. (e) Dyson orbitals’ isosurfaces.

Their singly occupied character can be concluded from d*I*/d*V* maps throughout a wider energy region: characteristic d*I*/d*V* patterns attributed to the SOMOs and their correlated singly unoccupied molecular orbitals (SUMOs) appeared at −700 and −300 meV and at 100 and 700 meV, respectively. Since all states detected around *E*_F_ lie close in energy, the molecular system is expected to exhibit a strong multiconfigurational character. Therefore, to identify and interpret the d*I*/d*V* maps, we computed the relevant Dyson orbitals^33^ using the natural orbitals obtained from complete active space configuration interaction (CASCI) calculations (see Methods and Supporting Information). In agreement with DFT, we obtained three Dyson orbitals accounting for electron addition and four for electron removal (Figure 3e), as expected for a ground state composed of three singly occupied and one doubly occupied state. In Figure 3d, we show the simulated d*I*/d*V* maps resulting from the computed Dyson orbitals, including a CO-functionalized tip, calculated with the PP-STM code.^34^ The maps reproduce the experimental d*I*/d*V* maps in great detail, further confirming the identification of three singly occupied states hosting the spin properties of the molecule.

The simulations indicate that **TTAT** maintains a neutral charge state on the electrophilic Au(111) surface. Kelvin probe force microscopy (KPFM) measurements provided in Figure S7 confirm that the molecule remains in a neutral state on the Au(111) substrate. This behavior contrasts with the cationic state found for the structurally similar molecule A5T, despite both molecules having the same spin imbalance and nullity.^14,15,35^ Although the precise factors driving charge transfer differences near chemical equilibrium would require further study, we speculate that the neutral stability of **TTAT** (compared to **TTAT** ^**+**^) is related to its enhanced aromaticity, evidenced, for example, by the larger number of Clar sextets. In contrast, the antiaromaticity of neutral A5T species accounts for its tendency to oxidize in Au(111).^14^

These observations suggest that **TTAT** has a spin ground state *S* = 3/2 on Au(111), with parallel spin alignment due to Hund’s exchange interactions among the three SOMOs.^36,37^ We thus attribute the zero-bias resonance in the STS spectra to an underscreened Kondo effect associated with this high-spin state. Nanographenes with spin above *S* = 1/2 generally exhibit partial Kondo screening on surfaces, leading to smaller zero-bias resonances,^9,38^ as seen in Figure 2a. Additional evidence for this underscreened Kondo effect is the resonance splitting under an external magnetic field (*B* = 2.7 T) as shown in Figure S8, supporting an *S* = 3/2 ground state for TTAT on Au(111).

### Triradical Character of TTAT

To rationalize the high-spin triradical ground state of **TTAT**, and the origin of the IETS features observed in the d*I*/d*V* spectra, we performed multiconfigurational calculations with the complete active space self-consistent (CASSCF) method. Considering a CAS­(7,10), we obtain that three natural orbitals (highlighted in the dashed box, Figure 4a) have an electron occupation close to 1, thus indicating the presence of three unpaired electrons in the ground state. In accordance with the DFT calculations and the experimental results, a natural orbital with A_1_ symmetry and centered on the N heteroatom appears with a larger electron occupation. As shown in the many-body energy levels in Figure 4b, the CASSCF calculations confirm the quartet (*S* = 3/2) ground state of **TTAT**, which accordingly is predominantly described by a single determinant,^39^ as depicted in Figure 4c.

**Figure 4:**
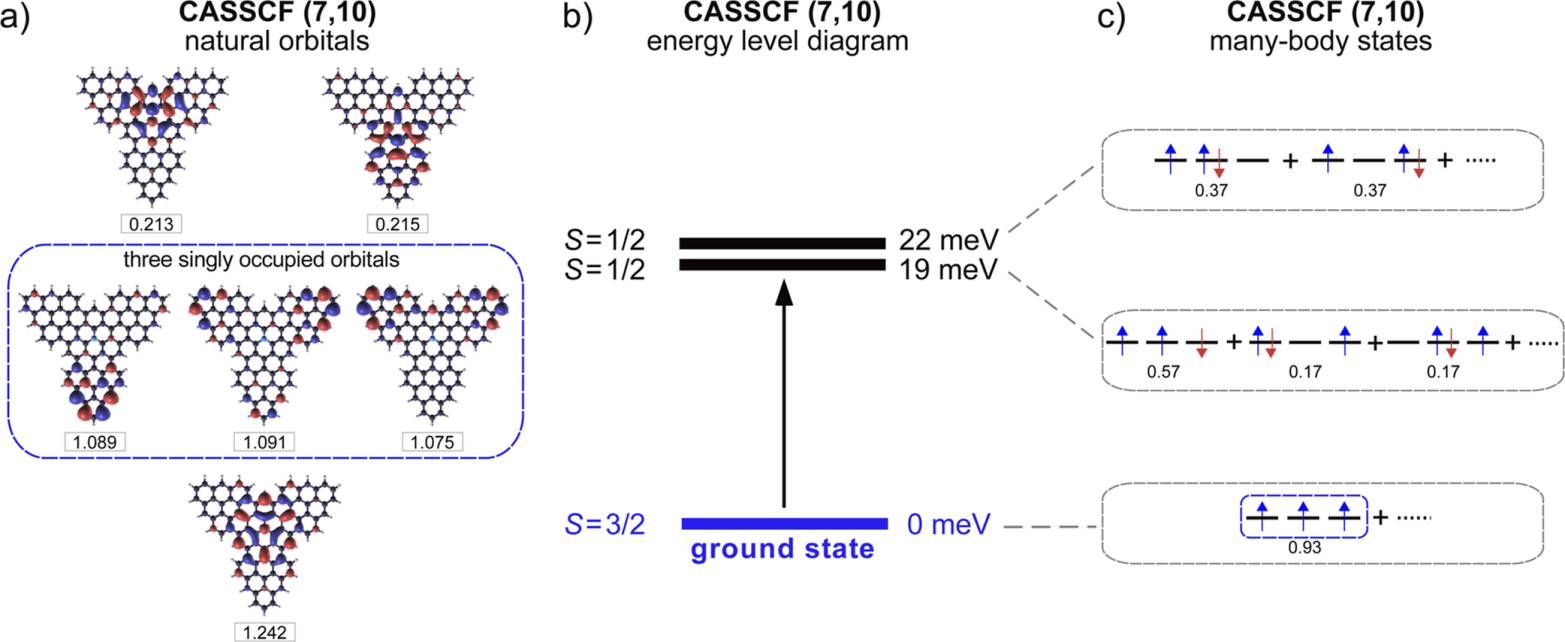
(a) Natural orbitals computed by CASSCF (7,10). The numbers at the bottom of each orbital indicate the electron occupation. We find three orbitals with occupation close to 1, spatially distributed over the triangulene edges. (b) Diagram representing the energy and the total spin of the many-body ground state and first two excited states of **TTAT**, as computed by CASSCF (7,10). (c) Schematic representation of the most relevant Slater determinants for each of the many-body states in (b), displaying the electronic occupation of the three natural orbitals highlighted in the dashed box in (a). The number below each Slater determinant refers to its weight in the corresponding many-body state.

The first two excited states are two nearly degenerate spin *S* = 1/2 levels found at 19 and 22 meV above the ground state. These values lie close to the experimental excitation gap obtained from the IETS steps (Δ ≈ 15 meV), supporting the identification of the inelastic spectral features with a spin excitation process from *S* = 3/2 to *S* = 1/2. These doublets are described by a linear combination of different Slater determinants with similar weights (Figure 4c), justifying multiconfigurational methods utilized in Figure 3.

The multiconfigurational nature of the doublet excited states is likely reflected in the experimental magnetic fingerprints shown in Figure 2b,c. To explain the spatial localization of the Kondo signal, we used the concept of Kondo orbitals recently introduced in ref 35 to describe the Kondo effect in open-shell polyradical molecular systems. In this framework, the Kondo orbitals are associated with scattering processes between molecular electrons and conduction electrons of the underlying metal, featuring antiferromagnetic exchange coupling. Using CASCI calculations, we obtained the set of Kondo orbitals shown in Figure S12, which we used for calculating the corresponding Kondo d*I*/d*V* map using the PP-STM code.^34^ The simulated d*I*/d*V* map shown in Figure 2d reproduces the shape and distribution of the experimental Kondo map, further confirming the interpretation of the Kondo signal as the fingerprint of a *S* = 3/2 spin state.

Similarly, the inelastic spin excitation from the quadruplet ground state to the two doublet excited states is represented by CASCI NTOs shown in Figure S11. The simulated spin excitation d*I*/d*V* map^34^ reported in Figure 2e was obtained by summing the contributions of the NTOs corresponding to the spin excitation to the two degenerate excited states. The excellent agreement with the experimental map of the IETS signal supports the origin of the inelastic signal as a quartet-doublet spin excitation.

The localization of spin fingerprints at the three vertices suggests that the open-shell character of **TTAT** could be described by three spatially localized radicals rather than a set of overlapping SOMOs. Using a maximally localized orbital basis set, we obtain a representation where each of the three unpaired electrons is mostly located at an individual triangulene corner, as displayed in the SOMOs in Figure 5a. We computed the spin–spin correlation $A_{ij}=\left\langle \hat{S_{i}\;}\hat{S_{j}\;}\right\rangle -\left\langle \hat{S_{i}\;}\right\rangle \left\langle \hat{S_{j}\;}\right\rangle$ for each pair of spins *i* and *j* in those maximally localized orbitals. The results, illustrated in Figure 5b, confirm the ferromagnetic coupling between the three unpaired spins in the ground state with the value of the spin–spin correlation *A*_*ij*_ = 0.22 au. In contrast to other ferromagnetic triradical systems^41^ with central moieties acting as spin couplers, in **TTAT** the SOMO states have a residual weight over the central aza-triangulene unit. Therefore, their small exchange is governed by the weak overlap of the three radicals.

**Figure 5:**
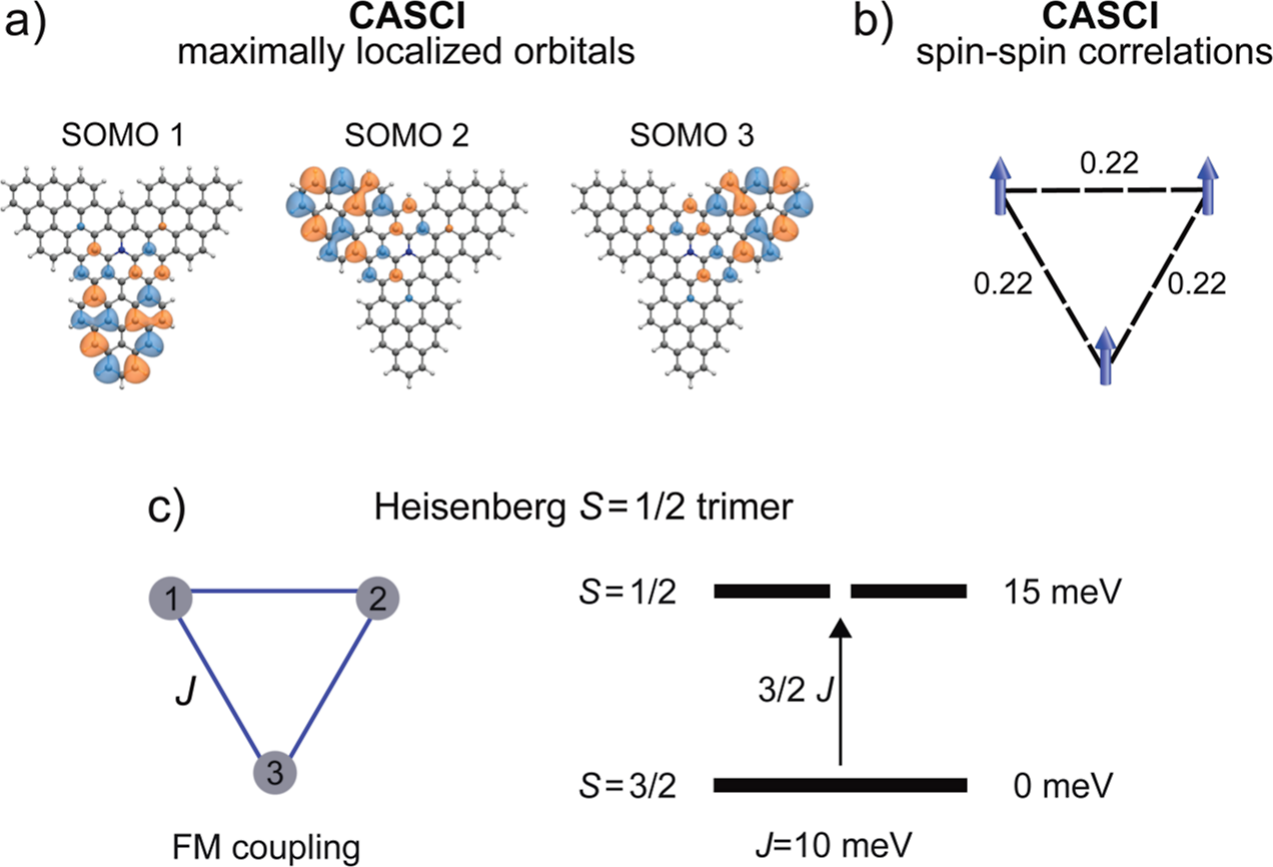
(a) Representation of the three singly occupied orbitals using a maximally localized basis set, which shows that each spin is mostly located on a triangulene corner. (b) Spin–spin correlation between each pair of spins computed using the orbital representation in (a). This picture describes the magnetic state of **TTAT** in terms of a symmetric Heisenberg ferromagnetic trimer, as illustrated in (c). According to this model, the experimental quartet-doublet energy gap of 15 meV corresponds to an exchange coupling *J* = 10 meV.^40^

This representation suggests the possibility of describing the triradical molecule **TTAT** as a symmetric Heisenberg *S* = 1/2 trimer.^40^ Considering an equal ferromagnetic coupling *J* between the three unpaired spins (Figure 5b), a Heisenberg spin model yields a ground state with total spin *S* = 3/2 and two degenerate doublets (*S* = 1/2) as the first excited states, similar to the results of the many-body CASSCF calculations. In the case of an equilateral Heisenberg trimer, the exchange *J* is given by $J=\frac{2}{3}\Delta E$ , where Δ*E* is the quartet-doublet energy difference. Therefore, considering the experimental excitation energy Δ*E* = 15 meV, we determine for **TTAT** an exchange coupling *J* = 10 meV, in good agreement with the value of 9 meV obtained from the fit to the STS data using the perturbative model by Ternes.^26^

## Conclusions

In summary, we have presented a polyradical aza-nanographene (**TTAT**) hosting three unpaired π electrons localized at the vertices of a triangle and coupled through symmetric ferromagnetic interactions. It was designed by combining well-known molecular building blocks, all-carbon and N-doped [3]­triangulenes, and fabricated via a combination of in-solution and on-surface synthesis. The detection of clear magnetic fingerprints in scanning tunneling spectroscopy (i.e., both a weak Kondo resonance and a IETS step-like feature) demonstrated the open-shell and polyradical character of the molecule on Au(111). Combining differential conductance spectra and orbital maps with DFT and advanced multiconfigurational CASSCF calculations, we revealed the presence of three radicals and their ferromagnetic alignment, resulting in an *S* = 3/2 (quartet) ground state. The many-body molecular states renormalize into three symmetric, weakly interacting radical states, forming a molecular trimer reminiscent of a Heisenberg system. We envision that the spin interactions within this triangular architecture can be further tuned by modifying its structure and composition through related synthesis processes. These resulting carbon-based platforms hold promise as potential candidates for the development of multispin quantum systems.

## Methods

The (111) surface of a gold single crystal was cleaned by several cycles of sputtering with Ne^+^ ions and subsequent annealing at *T* = 600 °C under ultrahigh vacuum conditions. The precursor of **TTAT** was prepared in solution as described in Figure 1 and in the Supporting Information (Section 1). The sublimation was achieved via the fast thermal heating of a Si wafer loaded with grains of compound **1**.

All measurements were conducted in a low-temperature STM at 5 K in ultrahigh vacuum conditions, except for those reported in Figure S8, performed in a commercial Joule–Thompson (JT) STM with a base temperature of 1.2 K. STM constant-current images were performed with a gold-coated tungsten tip or, when indicated in the text, with a CO-terminated tip. The BR STM and AFM images were always recorded using a CO-functionalized tip in the constant-height mode. The figures representing the experimental data were prepared using WSxM and SpectraFox software.^42,43^

Differential conductance spectra were recorded using a lock-in amplifier with a frequency *f* = 753 Hz (*f* = 887 Hz for the spectra in Figure S8). The modulation amplitude and current parameters are indicated in the captions of the respective figures.

The d^2^ *I*/d*V* ^2^ maps reported in Figure 2 were obtained through numerical differentiation of an *XY* matrix of d*I*/d*V* spectra taken over a region covering the molecule. Each d*I*/d*V* spectrum was recorded at a constant tip–sample height within the bias range (50, −50 mV) at each point of a 20 × 20 grid. After each individual spectrum measurement, the feedback loop was reactivated (with parameters *V* = 50 mV and *I* = 1 nA) before the tip was moved to the next position on the grid. After recording the whole matrix of d*I*/d*V* spectra, the differentiation of the d*I*/d*V* signal and the visualization of the spatial distribution of the resulting d^2^ *I*/d*V* ^2^ signal at different energies were performed using WSxM software.^42^ This procedure enabled the generation of d^2^ *I*/d*V* ^2^ maps at specific energies.^18^

The AFM BR image reported in Figure 1 and the KPFM measurements reported in Figure S7 were performed using a qPlus-type sensor with an eigenfrequency *f*_0_ = 30.72 kHz and a *Q*-factor of the order of 10^4^.^44^ The AFM was operated in the frequency modulation mode,^45^ with an oscillation amplitude *A* = 60 pm.

## Supplementary Material



## Notes

Solution synthesis details of molecular precursors, including NMR data of the synthesized compounds, complementary experimental results, and complementary theoretical methods and results, such as DFT orbitals, CASCI-based NTOs, and Kondo orbitals (PDF)

## References

[1] Li J., Sanz S., Corso M., Choi D. J., Peña D., Frederiksen T., Pascual J. I. (2019). Single spin localization and manipulation
in graphene open-shell nanostructures. Nat.
Commun..

[2] Mishra S., Beyer D., Eimre K., Kezilebieke S., Berger R., Gröning O., Pignedoli C. A., Müllen K., Liljeroth P., Ruffieux P., Feng X., Fasel R. (2020). Topological Frustration
Induces Unconventional Magnetism in a Nanographene. Nat. Nanotechnol..

[3] Gaita-Ariño A., Luis F., Hill S., Coronado E. (2019). Molecular Spins for
Quantum Computation. Nat. Chem..

[4] Yazyev O. V. (2010). Emergence
of Magnetism in Graphene Materials and Nanostructures. Rep. Prog. Phys..

[5] de
Oteyza D. G., Frederiksen T. (2022). Carbon-Based Nanostructures as a
Versatile Platform for Tunable π-Magnetism. J. Phys.: Condens. Matter.

[6] Clair S., de Oteyza D. G. (2019). Controlling
a Chemical Coupling Reaction on a Surface:
Tools and Strategies for On-Surface Synthesis. Chem. Rev..

[7] Ovchinnikov A. A. (1978). Multiplicity
of the Ground State of Large Alternant Organic Molecules with Conjugated
Bonds: (Do Organic Ferromagnetics Exist?). Theor.
Chim. Acta.

[8] Pavliček N., Mistry A., Majzik Z., Moll N., Meyer G., Fox D. J., Gross L. (2017). Synthesis
and characterization of
triangulene. Nat. Nanotechnol..

[9] Turco E., Bernhardt A., Krane N., Valenta L., Fasel R., Juríček M., Ruffieux P. (2023). Observation of the
Magnetic Ground State of the Two Smallest Triangular Nanographenes. JACS Au.

[10] Mishra S., Beyer D., Eimre K., Liu J., Berger R., Gröning O., Pignedoli C. A., Müllen K., Fasel R., Feng X., Ruffieux P. (2019). Synthesis
and Characterization
of π-Extended Triangulene. J. Am. Chem.
Soc..

[11] Su J., Telychko M., Hu P., Macam G., Mutombo P., Zhang H., Bao Y., Cheng F., Huang Z. Q., Qiu Z. (2019). Atomically
Precise Bottom-up Synthesis of π-Extended
[5]­Triangulene. Sci. Adv..

[12] Mishra S., Yao X., Chen Q., Eimre K., Gröning O., Ortiz R., Di Giovannantonio M., Sancho-García J. C., Fernández-Rossier J., Pignedoli C. A., Müllen K., Ruffieux P., Narita A., Fasel R. (2021). Large Magnetic
Exchange Coupling in Rhombus-Shaped Nanographenes with Zigzag Periphery. Nat. Chem..

[13] Wang T., Berdonces-Layunta A., Friedrich N., Vilas-Varela M., Calupitan J. P., Pascual J. I., Peña D., Casanova D., Corso M., de Oteyza D. G. (2022). Aza-Triangulene:
On-Surface Synthesis and Electronic and Magnetic Properties. J. Am. Chem. Soc..

[14] Vilas-Varela M., Romero-Lara F., Vegliante A., Calupitan J. P., Martínez A., Meyer L., Uriarte-Amiano U., Friedrich N., Wang D., Schulz F. (2023). On-Surface
Synthesis and Characterization of a High-Spin Aza-[5]-Triangulene. Angew. Chem., Int. Ed..

[15] Lawrence J., He Y., Wei H., Su J., Song S., Wania Rodrigues A., Miravet D., Hawrylak P., Zhao J., Wu J., Lu J. (2023). Topological Design and Synthesis of High-Spin Aza-Triangulenes without
Jahn–Teller Distortions. ACS Nano.

[16] Mishra S., Beyer D., Eimre K., Ortiz R., Fernández-Rossier J., Berger R., Gröning O., Pignedoli C. A., Fasel R., Feng X., Ruffieux P. (2020). Collective
All-Carbon
Magnetism in Triangulene Dimers. Angew. Chem.,
Int. Ed..

[17] Zheng Y., Li C., Xu C., Beyer D., Yue X., Zhao Y., Wang G., Guan D., Li Y., Zheng H. (2020). Designer
spin order in diradical nanographenes. Nat.
Commun..

[18] Hieulle J., Castro S., Friedrich N., Vegliante A., Lara F. R., Sanz S., Rey D., Corso M., Frederiksen T., Pascual J. I., Peña D. (2021). On-Surface
Synthesis and Collective Spin Excitations of a Triangulene-Based Nanostar. Angew. Chem., Int. Ed..

[19] Cheng S., Xue Z., Li C., Liu Y., Xiang L., Ke Y., Yan K., Wang S., Yu P. (2022). On-surface synthesis of triangulene
trimers via dehydration reaction. Nat. Commun..

[20] Du Q., Su X., Liu Y., Jiang Y., Li C., Yan K., Ortiz R., Frederiksen T., Wang S., Yu P. (2023). Orbital-symmetry
effects on magnetic exchange in open-shell nanographenes. Nat. Commun..

[21] Turco E., Wu F., Catarina G., Krane N., Ma J., Fasel R., Feng X., Ruffieux P. (2024). Magnetic Excitations in Ferromagnetically
Coupled Spin-1 Nanographenes. Angew. Chem.,
Int. Ed..

[22] Calupitan J. P., Berdonces-Layunta A., Aguilar-Galindo F., Vilas-Varela M., Peña D., Casanova D., Corso M., de Oteyza D. G., Wang T. (2023). Emergence of π-Magnetism in Fused Aza-Triangulenes: Symmetry
and Charge Transfer Effects. Nano Lett..

[23] Song S., Pinar Solé A., Matěj A., Li G., Stetsovych O., Soler D., Yang H., Telychko M., Li J., Kumar M. (2024). Highly Entangled Polyradical Nanographene with Coexisting
Strong Correlation and Topological Frustration. Nat. Chem..

[24] Fajtlowicz S., John P. E., Sachs H. (2005). On Maximum
Matchings and Eigenvalues
of Benzenoid Graphs. Croat. Chem. Acta.

[25] Wang W. L., Yazyev O. V., Meng S., Kaxiras E. (2009). Topological
Frustration
in Graphene Nanoflakes: Magnetic Order and Spin Logic Devices. Phys. Rev. Lett..

[26] Ternes M. (2015). Spin Excitations
and Correlations in Scanning Tunneling Spectroscopy. New J. Phys..

[27] de
la Torre B., Švec M., Foti G., Krejčí O., Hapala P., Garcia-Lekue A., Frederiksen T., Zbořil R., Arnau A., Vázquez H., Jelínek P. (2017). Submolecular Resolution by Variation of the Inelastic
Electron Tunneling Spectroscopy Amplitude and its Relation to the
AFM/STM Signal. Phys. Rev. Lett..

[28] Gross L., Mohn F., Moll N., Liljeroth P., Meyer G. (2009). The Chemical Structure of a Molecule
Resolved by Atomic Force Microscopy. Science.

[29] Ortiz R., Fernández-Rossier J. (2020). Probing local moments
in nanographenes
with electron tunneling spectroscopy. Prog.
Surf. Sci..

[30] Mishra S., Catarina G., Wu F., Ortiz R., Jacob D., Eimre K., Ma J., Pignedoli C. A., Feng X., Ruffieux P., Fernández-Rossier J., Fasel R. (2021). Observation of fractional edge excitations in nanographene spin chains. Nature.

[31] Krane N., Turco E., Bernhardt A., Jacob D., Gandus G., Passerone D., Luisier M., Juríček M., Fasel R., Fernández-Rossier J., Ruffieux P. (2023). Exchange Interactions
and Intermolecular Hybridization in a Spin-1/2 Nanographene Dimer. Nano Lett..

[32] Sandoval-Salinas M. E., Carreras A., Casanova D. (2019). Triangular
Graphene Nanofragments:
Open-Shell Character and Doping. Phys. Chem.
Chem. Phys..

[33] Ortiz J. V. (2020). Dyson-Orbital
Concepts for Description of Electrons in Molecules. J. Chem. Phys..

[34] Krejčí O., Hapala P., Ondráček M., Jelínek P. (2017). Principles
and simulations of high-resolution STM imaging with a flexible tip
apex. Phys. Rev. B.

[35] Calvo-Fernández A., Kumar M., Soler-Polo D., Eiguren A., Blanco-Rey M., Jelínek P. (2024). Theoretical model for multiorbital Kondo screening
in strongly correlated molecules with several unpaired electrons. Phys. Rev. B.

[36] Jacob D., Fernández-Rossier J. (2022). Theory of
Intermolecular Exchange
in Coupled Spin-1/2 Nanographenes. Phys. Rev.
B.

[37] Mishra S., Fatayer S., Fernández S., Kaiser K., Peña D., Gross L. (2022). Nonbenzenoid High-Spin
Polycyclic Hydrocarbons Generated by Atom
Manipulation. ACS Nano.

[38] Li J., Sanz S., Castro-Esteban J., Vilas-Varela M., Friedrich N., Frederiksen T., Peña D., Pascual J. I. (2020). Uncovering the Triplet Ground State of Triangular Graphene
Nanoflakes Engineered with Atomic Precision on a Metal Surface. Phys. Rev. Lett..

[39] Krylov A. I. (2005). Triradicals. J. Phys. Chem. A.

[40] Haraldsen J. T., Barnes T., Musfeldt J. L. (2005). Neutron
Scattering and Magnetic Observables
for *S* = 1/2 Molecular Magnets. Phys. Rev. B.

[41] Zhang H., Pink M., Wang Y., Rajca S., Rajca A. (2022). High-Spin *S* = 3/2
Ground-State Aminyl Triradicals: Toward High-Spin
Oligo-Aza Nanographenes. J. Am. Chem. Soc..

[42] Horcas I., Fernández R., Gómez-Rodríguez J. M., Colchero J., Gómez-Herrero J., Baro A. M. (2007). WSXM: A
software for scanning probe microscopy and a tool for nanotechnology. Rev. Sci. Instrum..

[43] Ruby M. (2016). SpectraFox:
A free open-source data management and analysis tool for scanning
probe microscopy and spectroscopy. SoftwareX.

[44] Giessibl F. J. (1998). High-Speed
Force Sensor for Force Microscopy and Profilometry Utilizing a Quartz
Tuning Fork. Appl. Phys. Lett..

[45] Albrecht T. R., Grütter P., Horne D., Rugar D. (1991). Frequency Modulation
Detection Using High- *Q* Cantilevers for Enhanced
Force Microscope Sensitivity. J. Appl. Phys..

