# Supplementary material for: On-Surface Synthesis of a Ferromagnetic Molecular Spin Trimer
Source: J Am Chem Soc. 2025 May 30;147(23):19530–8. doi: 10.1021/jacs.4c15736 (PMC12164331; doi:10.1021/jacs.4c15736)
Supplement: Supplementary file 1 [file ja4c15736_si_001.pdf]

# Supporting Information: On-surface Synthesis of a Ferromagnetic Molecular Spin Trimer

Alessio Vegliante,[†,∇] Manuel Vilas-Varela,[‡,∇] Ricardo Ortiz,[¶] Francisco Romero Lara,[†] Manish Kumar,[§] Lucía Gómez-Rodrigo,[†,‡] Stefano Trivini,[†,‖] Fabian Schulz,[†] Diego Soler-Polo,[§] Hassan Ahmoum,[†] Emilio Artacho,[†,⊥,¶,#] Thomas Frederiksen,[¶,⊥] Pavel Jelínek,[§,@] Jose Ignacio Pascual,[*,†,⊥] and Diego Peña[*,‡,△]

†*CIC nanoGUNE-BRTA, 20018 Donostia-San Sebastián, Spain*
‡*Centro Singular de Investigación en Química Biolóxica e Materiais Moleculares (CiQUS) and Departamento de Química Orgánica, Universidade de Santiago de Compostela, 15782 Santiago de Compostela, Spain*
¶*Donostia International Physics Center (DIPC), 20018 Donostia-San Sebastián, Spain*
§*Institute of Physics, Czech Academy of Sciences, 16200 Prague, Czech Republic*
‖*Materials Physics Center (CFM-MPC), E-20018 Donostia-San Sebastián, Spain*
⊥*Ikerbasque, Basque Foundation for Science, 48013 Bilbao, Spain*
#*Theory of Condensed Matter, Cavendish Laboratory, University of Cambridge, CB3 0HE Cambridge, United Kingdom*
@*Czech Advanced Technology and Research Institute (CATRIN), Palacký University Olomouc, 77900 Olomouc, Czech Republic*
△*Oportunius, Galician Innovation Agency (GAIN), 15702 Santiago de Compostela, Spain*
∇*Contributed equally to the work*

E-mail: ji.pascual@nanogune.eu; diego.pena@usc.es

# Contents

**1 Synthetic details** **S-2**
1.1 Solution Synthesis of Molecular Precursors . . . . . S-2
1.2 Synthesis of the TTAT Precursor . . . . . S-2
1.3 NMR Data . . . . . S-2

**2 Complementary experimental data** **S-4**

**3 Complementary theoretical methods and results** **S-7**

**References** **S-13**

# 1 Synthetic details

## 1.1 Solution Synthesis of Molecular Precursors

Starting materials were purchased reagent grade from TCI and Sigma-Aldrich and used without further purification. Trimethyl 2,2',2"-nitrilotribenzaldehyde (**2**) was obtained following reported procedures.[S1] Organolithium reagent **3** was prepared by treatment of the corresponding bromide[S2] with n-butyllithium. All reactions were carried out in flame-dried glassware under an inert atmosphere of purified Ar using Schlenk techniques. Thin-layer chromatography (TLC) was performed on Silica Gel 60 F-254 plates (Merck). Column chromatography was performed on silica gel (40-60 µm). NMR spectra were recorded on a Bruker Varian Inova 500 spectrometer.

## 1.2 Synthesis of the TTAT Precursor

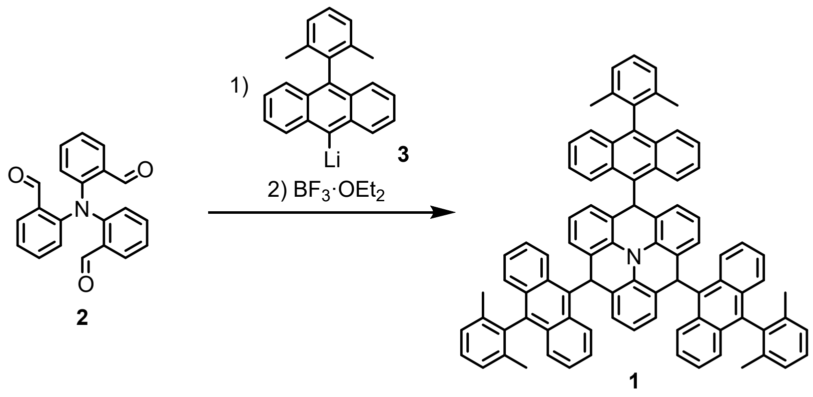


Figure S1: Synthesis of the **TTAT** precursor, compound **1**.

Over a solution of organolithium derivative **3** (1.74 mmol, 5.00 equiv) in THF (30 mL), aldehyde **2** (115 mg, 0.35 mmol) was added at 0 ºC. After addition, the mixture was allowed to reach room temperature and stirred for 16 h. MeOH (2 mL) was then added, and the solvents were evaporated under reduced pressure. The residue was redissolved in $CH_2Cl_2$ (15 mL) and $BF_3{\cdot}OEt_2$ (1.00 mL) was added at 0 ºC. After addition, the mixture was stirred for 20 min and then poured into an aqueous NaOH solution (0.50 M, 40.0 mL). After separation of phases, the aqueous fraction was extracted with $CH_2Cl_2$ (2 x 15 mL). The combined organic fractions were dried over anhydrous $Na_2SO_4$, filtered, and evaporated under reduced pressure. The residue was purified by column chromatography ($SiO_2$; hexane:$CH_2Cl_2$, 4:1), affording compound 1 (85 mg, 38%) as a yellow solid.

## 1.3 NMR Data

**$^1$H NMR (500 MHz, $CDCl_3$)** $\delta$: 8.69 – 8.59 (m, 3H), 7.55 – 7.48 (m, 3H), 7.47 – 7.36 (m, 5H), 7.32 – 7.26 (m, 3H), 7.21 – 7.14 (m, 5H), 6.52 – 6.47 (m, 2H), 6.43 (d, J = 7.7 Hz, 1H), 6.36 (t, J = 7.7 Hz, 1H), 1.73 (s, 3H), 1.71 (s, 3H), 1.63 (s, 3H) ppm.

**$^{13}$C NMR (125 MHz, $CDCl_3$)** $\delta$: 138.72 (C), 138.48 (C), 138.20 (C), 138.13 (C), 137.91 (C), 137.87 (C), 136.76 (C), 136.71 (C), 133.13 (C), 132.92 (C), 132.76 (C), 132.22 (C), 132.01 (C), 131.99 (C), 131.10 (C), 131.04 (C), 129.23 (C), 129.12 (C), 129.10 (C), 128.25 (CH), 128.13 (CH), 127.83 (CH), 127.64 (CH), 127.61 (CH), 127.25 (CH), 127.23 (CH), 127.21 (CH), 127.17 (CH), 127.12 (CH), 127.03 (CH), 126.05 (C), 125.65 (C), 125.62 (CH), 125.58 (C), 125.43 (CH), 125.40 (CH), 125.34 (CH), 125.22 (CH), 125.19 (CH), 123.89 (CH), 123.72 (CH), 123.38 (CH), 123.29 (CH),

39.16 (CH), 20.30 ($CH_3$), 20.27 ($CH_3$), 20.18 ($CH_3$) ppm. **MS (APCI)** m/z (%): 1122 (M+1, 93), 1019 (48), 841 (58). **HRMS (APCI)**: $C_{87}H_{64}N$; calculated: 1122.5033, found: 1122.5011.

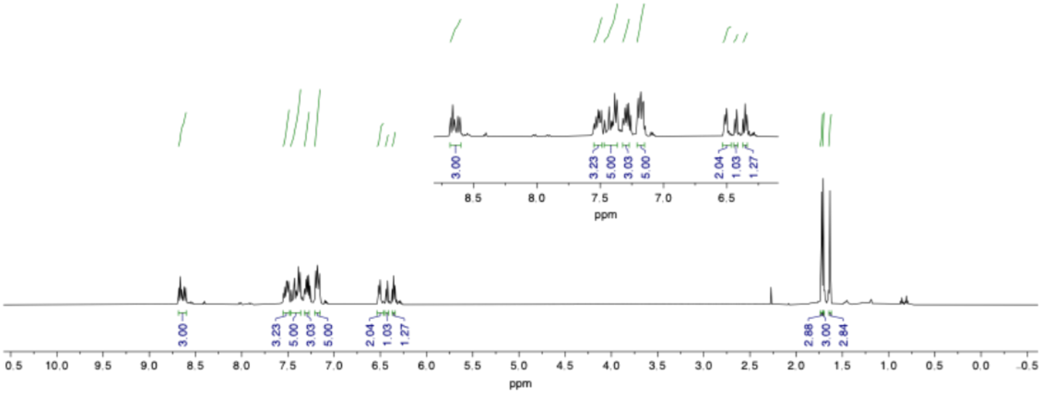


Figure S2: $^1$H NMR (500 MHz, $CDCl_3$) spectrum of compound **1**.

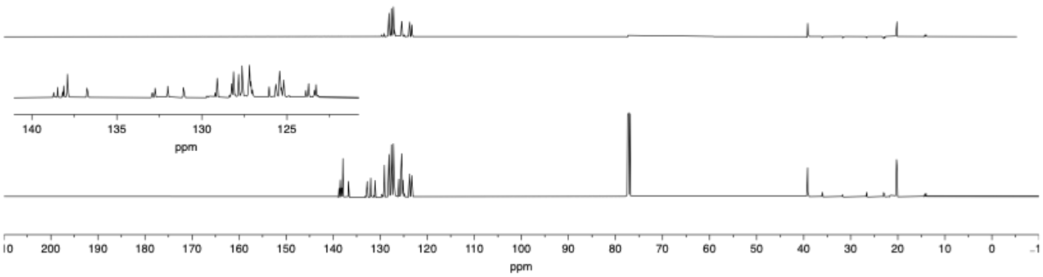


Figure S3: $^{13}$C NMR-DEPT (125 MHz, $CDCl_3$) spectra of compound **1**.

## 2 Complementary experimental data

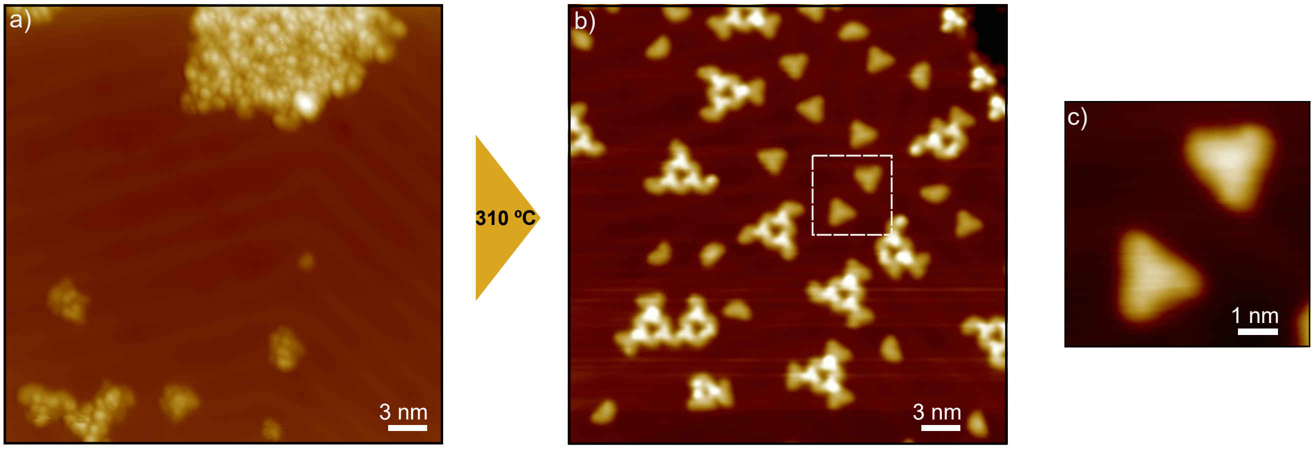


Figure S4: a) STM constant-current overview image ($V = 1$ V; $I = 30$ pA) after deposition of the molecular precursor on Au(111) at room temperature, showing mostly three-dimensional molecular clusters and aggregates. b-c) After annealing at 310°C, smaller domains and individual molecules are observed. The presence of rounded, protruding corners indicate that the isolated molecules have not undergone the cyclodehydrogenation reaction yet, and thus still retain their methyl groups ($V = 1$ V; $I = 30$ pA). A further annealing at 330°C is needed in order to fully activate the on-surface reaction and generate **TTAT** (Fig. 1 in the main text).

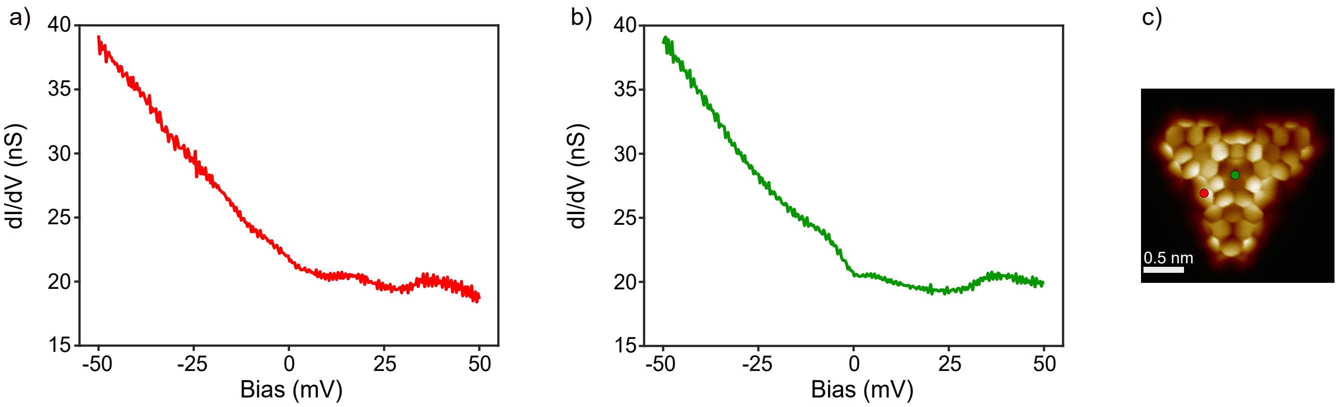


Figure S5: Low-bias $dI/dV$ spectra measured with a CO-functionlized tip on the gulf area between the external triangulenes (a) and on the central N atom (b), as indicated in the bond-resolved constant-height image ($V = 5$ mV) in c). The Kondo and spin excitation features measured on the triangulene corners (Fig. 2 in the main text) are absent in these regions, while we observe here a conductance increase due the onset of the molecular orbital at $V = -100$ mV. Spectroscopy parameters: $V = 50$ mV, $I = 1$ nA, $V_{mod} = 2$ mV.

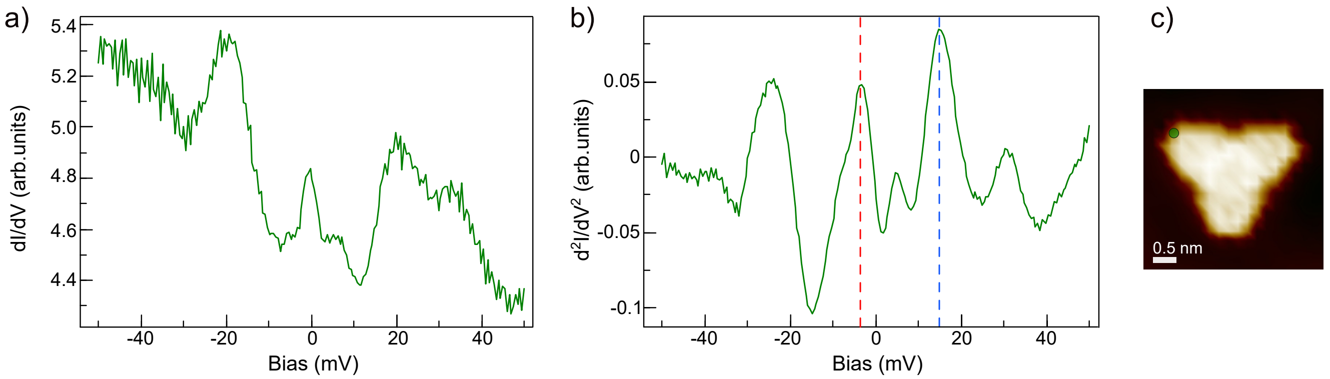


Figure S6: Single $dI/dV$ spectrum (a) and correspondent $d^2I/dV^2$ plot (b) extracted from a $dI/dV$ grid in the position indicated in c). Alongside the Kondo and spin excitation features, we can observe inelastic steps around $V = \pm 5$ mV and $V = \pm 35$ mV that originate from vibrational modes of the CO molecule attached to the tip. The red and blue dashed lines in (c) indicate the peaks (at $V = -3.3$ mV and $V = 15$ mV) that were selected to map the spatial distribution of the Kondo and the spin excitation feature, respectively, as reported in Fig. 2 in the main text. Parameters: $V = 50$ mV, $I = 1$ nA, $V_{mod} = 2$ mV.

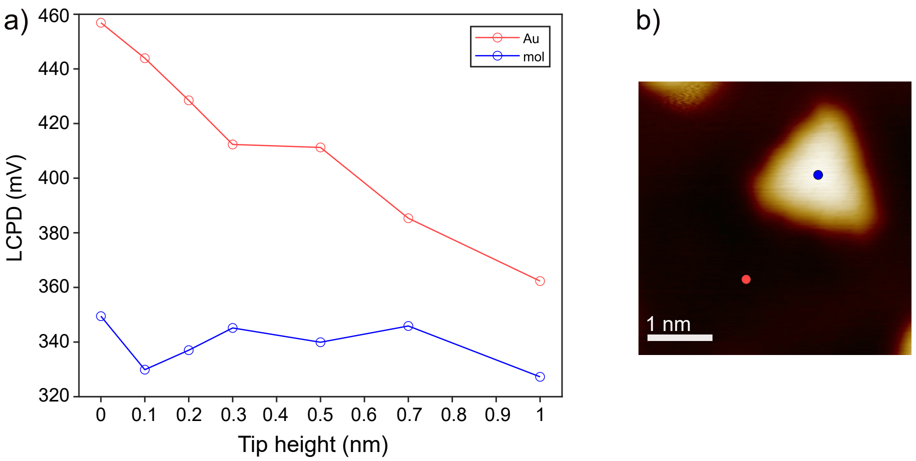


Figure S7: Kelvin probe force microscopy (KPFM) measurements on **TTAT**. a) Local contact potential difference (LCPD) values measured at different tip-molecule distances on the molecule and on the bare Au(111), as indicated in the constant-current STM image in (b). The 0 distance corresponds to the setpoint ($I = 30$ pA; $V = 200$ mV) over the centre of the molecule. For each tip height, we first measure a $\Delta f(V)$ spectrum (frequency shift as a function of the tip-sample bias) in the range (-0.1V, 0.9V) over the molecule, then we move the tip to the bare Au (with the feedback loop open) and record the same spectrum at the same height, and determine the LCPD for each position from the vertex of the KPFM parabola.[S3] We observe that the LCPD values on the molecule are always more negative than those measured on the substrate. However, this lower LCPD value is not due to a net charge of the molecule, but to the so-called *push-back* effect (also known as pillow effect). This effect arises when a molecule (or an atom) is adsorbed on a metallic surface, and refers to the compression of the electron density of the metal that leaks into the vacuum by the adsorbate. It reduces the local work function of the surface[S4] and gives rise to a small shift of the LCPD towards more negative bias voltages.[S5] This is a general characteristic of metal-organic interfaces and independent of any charge transfer between adsorbate and metal surface. If, in addition, there are changes in the adsorbate´s charge state, i.e., net charges on the molecule, this would further modify the local work function. In that case, the shift of the LCPD would be much more pronounced (typically a few hundred meV at close tip-sample distances) and would show a strong dependence on the tip-sample distance.[S3,S6,S7] Because these effects are not observed in our LCPD measurements, we conclude that charge transfer between TTAT and the Au(111) surface is negligible.

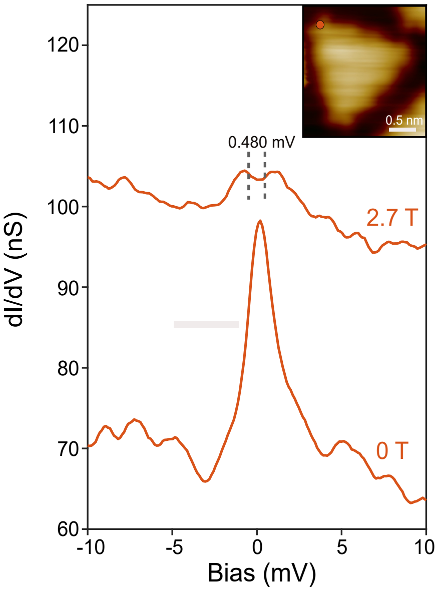


Figure S8: Comparison of low-bias $dI/dV$ spectra measured in the position indicated in the inset, in the absence of external magnetic field, and at 2.7 T field. The Kondo resonance shows a splitting in the presence of the magnetic field, which can be interpreted as an indication of an underscreend Kondo effect. An underscreened Kondo resonance is expected to undergo a splitting as soon as the Zeeman energy $(g\mu_B B)$ becomes greater than the thermal broadening $(k_B T)$, which in our experiment $(T = 1.2$ K) corresponds to a field $B \approx 1$T.[S8] A fully screened $S = 1/2$ Kondo resonance, on the other hand, would not display any splitting at this relatively low field, as we showed in previous works.[S1,S9] This observation suggests a high-spin ground state (i.e., larger than 1/2), but does not allow determining the exact total spin number.[S1] However, considering the neutral charge state of **TTAT** on Au(111), deduced from the dI/dV maps of Fig. 3 and the KPFM data of Fig. S7, the measurements are consistent with a S=3/2 ground state.[S1,S9] Parameters: $V = 10$ mV, $I = 1$ nA, $V_{mod} = 0.5$ mV. These measurements were performed at $T = 1.2$ K. The markers used to determine the energy splitting of the resonance are placed at the points of highest slope within the split peak, following the procedure used in our previous works.[S1,S9] It is important to note, however, that estimating the exact value of the energy splitting is not straightforward for weak magnetic fields and in the presence of significative overshoots (third order effects).[S10]

# 3 Complementary theoretical methods and results

**DFT simulations:**

First-principles calculations were performed using density-functional theory (DFT) implemented in the SIESTA code using the PBE generalised-gradient approximation. A double-zeta polarised (DZP) basis set was used as generated by a 50 meV energy shift along with a split norm of 0.15, except for H, for which the splitnorm was of 0.5 [1,2]. Core electrons were replaced by norm-conserving Troullier-Martins pseudopotentials [5] with cutoff radii of 1.54 Å for the s, p, d, and f channels of C; and 1.25 Å for H. The molecule was allowed to relax up to a force tolerance of 10 meV/Å.

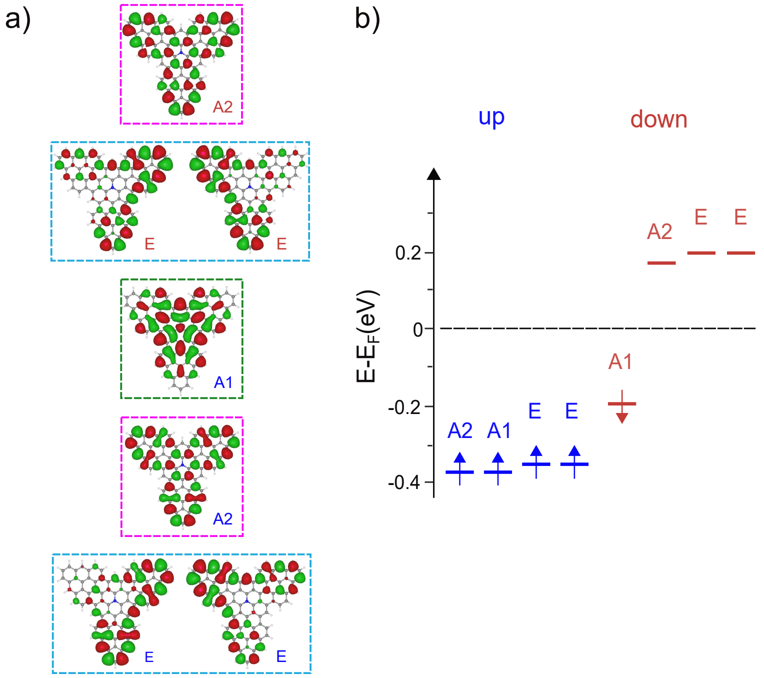


Figure S9: a) Molecular orbital isosurfaces corresponding to the single-particle states shown in the energy level diagram in b) obtained from spin-polarized DFT calculations. The labels refer to the symmetry of each orbital, defined in terms of irreducible representations of the $C_{3v}$ symmetry point group. The singly occupied orbitals are three: two degenerate orbitals with E symmetry, and one with A2 symmetry. The only orbital with the highest intensity on the N site is the A1, which is fully occupied.

**CASSCF calculations:**

We performed CASSCF calculations on **TTAT** with the ORCA 5.0.2 package.[S11] First, the geometry was relaxed at the DFT level, with the PBE density functional and def2-SVP basis set. An auxiliary basis set was automatically generated with the AutoAux keyword. The resulting DFT orbitals and optimized geometry were then used as input for CASSCF, where the Complete Active Space consisted in 7 electrons fluctuating in 10 orbitals (CAS(7,10)). The optimized natural orbital with most fractional occupation had 1.82 electrons, while the natural orbital with less fractional occupation had 0.05 electrons.

**CASCI calculations:**

The molecular geometries were optimized in quartet states using density functional theory (DFT), as implemented in the FHI-AIMS software package,[S12] employing the PBE0 hybrid functional.[S13] In these calculations, the Tkatchenko-Scheffler method was used to account for Van der Waals interactions. Given the open-shell and multi-radical nature of the molecules under investigation, the complete active space configuration interaction (CASCI) method was employed to obtain an

accurate description of the wave function and electronic energies. One- and two-electron integrals are constructed in the basis of molecular orbitals around the fermi energy were derived using the quantum chemistry software ORCA[S11] using the orbitals from the restricted open-shell Hartree Fock (ROHF).

$$t_{ij} = \int \phi_i(\mathbf{r}) \left( -\frac{\hbar}{2m} \nabla^2 + V(\mathbf{r}) \right) \phi_j(\mathbf{r}) d^3\mathbf{r} \tag{1}$$

and

$$\mathcal{V}_{ijkl} = \frac{1}{4\pi\epsilon_0} \int \frac{\phi_i(\mathbf{r})\phi_j(\mathbf{r}')\phi_k(\mathbf{r}')\phi_l(\mathbf{r})}{|\mathbf{r}-\mathbf{r}'|} d^3\mathbf{r} d^3\mathbf{r}', \tag{2}$$

where the indices $i, j, k, l$ denote molecular orbitals, extending across the set of orbitals chosen as the active space (CAS(11,11) for aza-triangulene and CAS(12,12 for full carbon analogue of aza-triangulene)). The one-electron potential $V(\mathbf{r})$ in Eq. 1 encompasses the ionic potentials and the contributions from electrons in the occupied inactive orbitals, that is,

$$V(\mathbf{r}) = \frac{1}{4\pi\epsilon_0} \sum_{\gamma} \frac{eZ_\gamma}{|\mathbf{R}_\gamma - \mathbf{r}|} + \sum_{\lambda} \int \frac{|\phi_\lambda(\mathbf{r}')|^2}{|\mathbf{r}-\mathbf{r}'|} d^3\mathbf{r},$$

where index $\gamma$ runs over the nuclei, $Z_\gamma$ is the charge of the nuclei and the index $\lambda$ runs over occupied inactive molecular orbitals given by $\phi_\lambda(\mathbf{r})$, which are always doubly-occupied in the possible Slater determinants of our calculation. Subsequently, we construct the many-body *ab initio* molecular Hamiltonian $\hat{\mathcal{H}}_{\text{CAS}}$ using these coefficients:

$$\hat{\mathcal{H}}_{\text{CAS}} = \sum_{i,j,\sigma} t_{ij} \hat{c}^\dagger_{i\sigma} \hat{c}_{j\sigma} + \sum_{i,j,k,l,\sigma,\sigma'} \mathcal{V}_{ijkl} \hat{c}^\dagger_{i\sigma} \hat{c}^\dagger_{j\sigma'} \hat{c}_{k\sigma'} \hat{c}_{l\sigma}. \tag{3}$$

where $\hat{c}_{i\sigma}$ ($\hat{c}^\dagger_{i\sigma}$) denotes the annihilation (creation) operator of electron with spin $\sigma$ in $i$-th orbital. The full many-body Hamiltonian Eq. (3) was diagonalized in our in-house code to obtain the many-body wave function $\Psi$ given by linear combination of Slater determinants.

To get the number of unpaired electrons in the molecule, we have constructed the one-particle density matrix, $\rho_{ij} = \langle \Psi | \hat{c}^\dagger_i \hat{c}_j | \Psi \rangle$ from the ground state many-body CASCI wavefunction. Natural orbitals are eigenvectors obtained from diagonalization of the one-particle reduced density matrix $\rho$, which positive eigenvalues represent the occupations of the natural orbitals. The natural orbitals whose occupations have fractional values significantly different from integer values of 2 or 0 contribute to the number of unpaired electrons in the molecule. Fig. S10b shows the natural orbitals obtained for the aza-triangulene molecule.

**Natural Transition Orbitals (NTO):**

To simulate the $dI/dV$ maps corresponding to IETS spin excitation maps, we have calculated the Natural Transition Orbitals (NTOs),[S14] which correspond to the electronic transition density matrix of single spin flip process from the quartet ground state to the doublet excited states. NTO orbitals are obtained from the diagonalization of the matrix $TT^\dagger$, where the matrix $T$ is given by elements

$$T_{jk} = \langle \Psi_{\text{doublet}} | \hat{c}^\dagger_{j\uparrow} \hat{c}_{k\downarrow} | \Psi_{\text{quartet}} \rangle,$$

where the indices $j, k$ run over the orbitals of active space. We have constructed the $T_{tk}$ matrices from the many-body ground and first excited state CASCI wavefunctions and Fig. S11 displays the calculated NTOs with corresponding amplitudes.

**Kondo Orbitals (KO):**

Kondo orbitals are calculated by diagonalizing the Hamiltonian derived from the multi-channel Anderson model, which considers the many-body multiplet structure of molecules obtained from

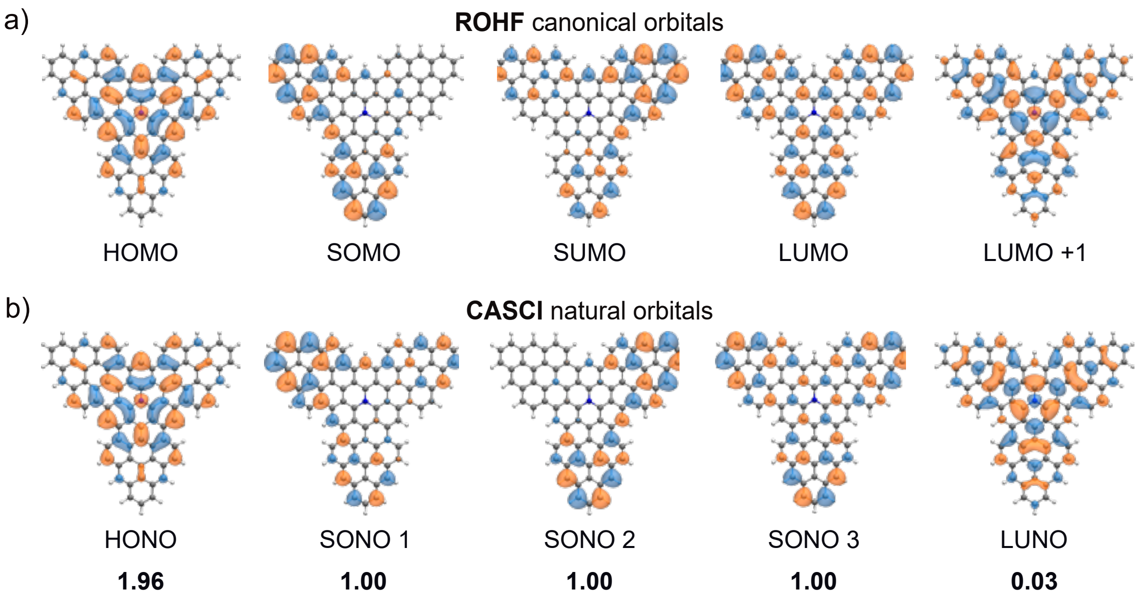


Figure S10: a) Restricted open-shell Hartree-Fock (ROHF) orbitals and (b) complete active space configuration interaction (CASCI) natural orbitals of **TTAT**. The numbers below the natural orbitals indicate their fractional electronic occupation. As for the CASSCF calculations reported in the main text, three singly occupied natural orbitals (SONO) are obtained.

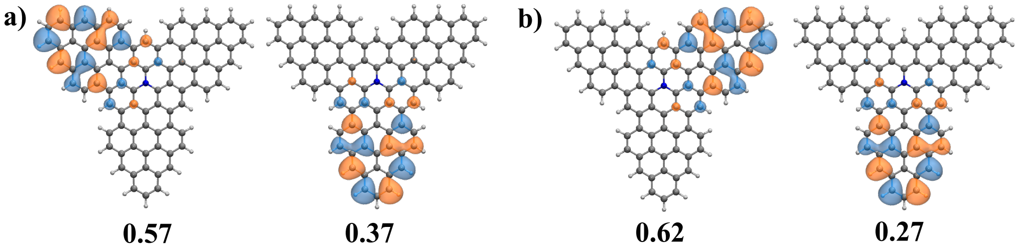


Figure S11: NTOs for the spin excitation from quartet ground state to a) first doublet and b) second doublet that is degenerate.

the CASCI calculation for the neutral ground state and virtual charge states as described in the Ref.[S15] For the calculation of scattering amplitude corresponding to virtual processes that occur in the intermediate charge state with one more and one less electron of the molecular state, we have taken the five low-energy multiplets in charge states. The amplitude from the high-energy multiplets is negligible because the reduced Lehmann amplitude is divided by the energy difference between the neutral state and the energy of the charge multiplet.

**Dyson Orbitals:** To accurately interpret differential conductance ($dI/dV$) maps for molecules exhibiting significant multireference character, it is necessary to move beyond the single-determinant molecular orbital framework typically provided by density functional theory (DFT) calculations. In this context, we have constructed the Dyson orbitals to simulate the $dI/dV$ maps, as they are more relevant for the single-electron removal and addition processes in STM.[S16,S17] Dyson orbitals corresponding to the spatial negative ion resonance (NIR) and positive electron affinity (PEA) are constructed as

$$\phi_{\mathrm{NIR}}(\mathbf{r}) = \langle \Psi^{N-1} | \hat{c}(\mathbf{r}) | \Psi^{N} \rangle. \tag{4}$$

$$\phi_{\mathrm{PEA}}(\mathbf{r}) = \langle \Psi^{N+1} | \hat{c}^{\dagger}(\mathbf{r}) | \Psi^{N} \rangle. \tag{5}$$

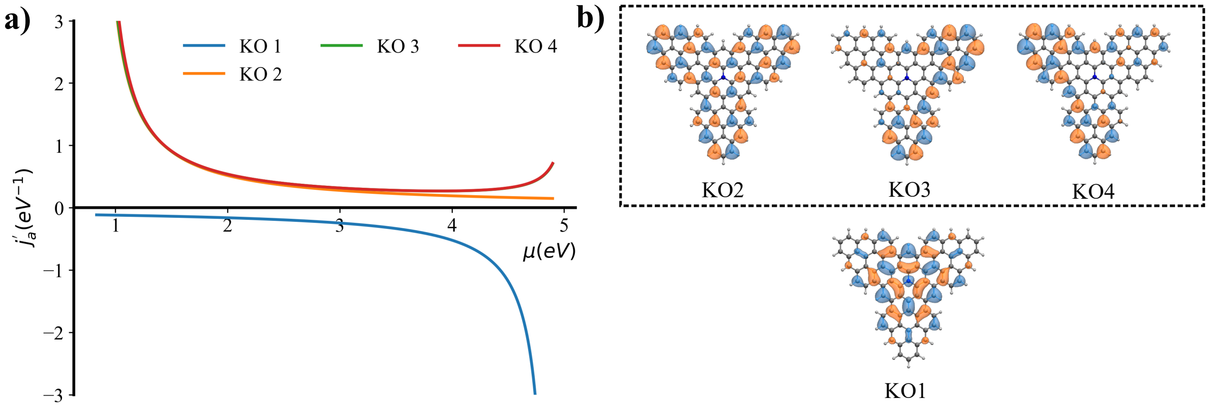


Figure S12: Results from the multi-orbital Kondo analysis, used to simulate the Kondo spatial distribution map presented in the main text. (a) Coupling constants $j_a$ computed as a function of the chemical potential for each Kondo orbital (KO). (b) Orbital isosurfaces of the four KOs with non-zero coupling to the conduction electrons of the substrate at a chemical potential of 3 eV. Three of these orbitals (highlighted in the dashed box) correspond to channels with antiferromagnetic coupling ($j_a > 0$) to the electron bath and therefore are involved in the many-body Kondo screening process.

where $\Psi^N$, $\Psi^{N-1}$, and $\Psi^{N+1}$ are the many-body wavefunction obtained from the CASCI calculations for the N, N-1 and N+1 electrons respectively.

Dyson orbitals provide a more comprehensive description of the electronic states involved in these tunneling events, capturing the electron removal/addition mechanisms during STS measurements. Fig.s S13 and S14 displays calculated Dyson orbitals and their amplitudes for removal/addition of a single electron.

Theoretical $dI/dV$ maps of NTOs and Kondo orbitals were calculated by the Probe Particle Scanning Probe Microscopy (PP-SPM) code[S18] for a CO-like tip. Heat maps are used to show the spatial current in the constant height mode for the theoretical dI/dV, where brighter colors represent the higher current values while black means no current. For NTOs we have chosen the tip composed of pxpy (90%) and s (10%) orbitals while for Kondo orbitals the tip is composed of pxpy (85%) and s (15%) orbitals to match the experimental $dI/dV$ maps.

It is important to notice that, although these calculations do not directly include the surface, they consider variations of the charge state of the molecule, as discussed in the previous sections. Additionally, in our simulations, we assume that all the Kondo orbitals have identical Kondo coupling with the metal substrate, which is a good approximation since all the states are $\pi$ orbitals with similar localization.

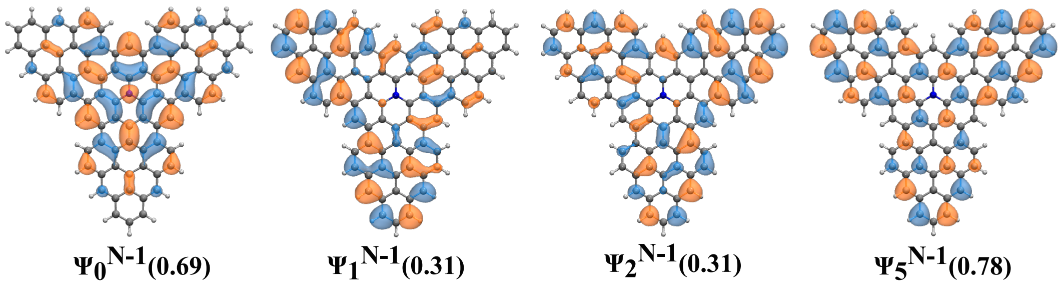


Figure S13: Dyson orbitals for the process of removal of one electron with the norm of the wavefunction below.

**Fitting to a spin model:**

We performed the CASCI calculation in the basis of maximally localized orbitals (see Fig. 5a of

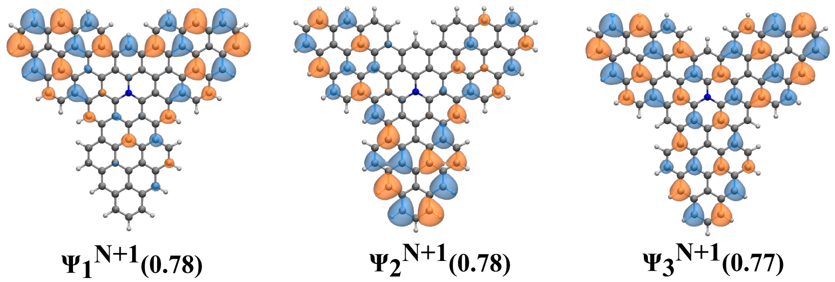


Figure S14: Dyson orbitals for the process of adding one electron with the norm of the wavefunction below.

the main text). In such representation, the wavefunctions of the ground and the excited states do not contain any fluctuations to states with doubly-occupied orbitals due to large Coulomb on-site interaction given by maximally localized character of orbitals.

Let us index with $j$ the three maximally localized orbitals. Then we construct the spin operators of the sites $j$: $\hat{\vec{S}}_j = \sum_{\sigma\sigma'} \vec{P}_{\sigma\sigma'} \hat{c}^\dagger_{j\sigma'} \hat{c}_{j\sigma}$, where $\vec{P}_{\sigma\sigma'}$ is the vector of components $\sigma, \sigma'$ of the $\frac{1}{2}$ Pauli matrices. We can then compute the spin correlations on this representation:

$$\mathbb{C}_{jk} = \langle \Psi | \hat{\vec{S}}_j \cdot \hat{\vec{S}}_k | \Psi \rangle - \langle \Psi | \hat{\vec{S}}_j | \Psi \rangle \cdot \langle \Psi | \hat{\vec{S}}_k | \Psi \rangle, \tag{6}$$

where $\Psi$ is the quartet ground state obtained from the CASCI calculation.

Further, as seen in the many-body spectra, there are two degenerate duplets for the diagonalization of the hamiltonian in the sector $S_z = \frac{1}{2}$.

Therefore, our system is well described by the ferromagnetic Heisenberg model:

$$\hat{H} = J \left( \hat{\vec{S}}_1 \cdot \hat{\vec{S}}_2 + \hat{\vec{S}}_1 \cdot \hat{\vec{S}}_3 + \hat{\vec{S}}_2 \cdot \hat{\vec{S}}_3 \right),$$

where $J = 10$ meV to reproduce the spin excitation and the homogeneous spin correlations. Notice that the identical coupling for all pairs of spins means that, in the duplet excited state, the trimer behaves as a frustrated magnet.

**Comparison with the all-carbon analogue:**

In the case of the aza-triangulene, CAS(11,11) calculations determine three unpaired electrons located mainly on the three external triangulenes, where the spin density is located (Fig. S15a). In contrast, in the fully carbon analogue, CAS(12,12) calculations predict four unpaired electrons (as anticipated in the scheme in Fig. 1a in the main text) that are delocalized throughout the molecule (Fig. S15b): three unpaired electrons come from the three external triangulenes, and one unpaired electron is localized in the central part.

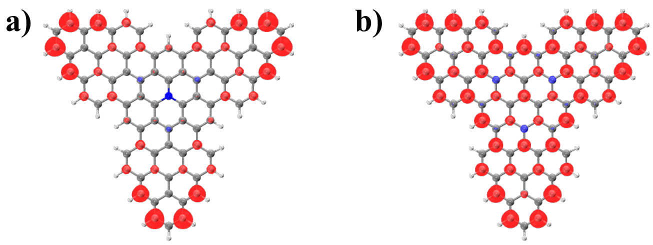


Figure S15: Calculated spin density maps for the (a) aza-triangulene molecule obtained from CAS(11,11) and (b) fully carbon analogue obtained from CAS(12,12) calculations.

From the occupation of the many-body natural orbitals obtained from CAS(11,11), it is clear that **TTAT** displays three orbitals with an occupation of 1, primarily located on the external triangulenes (Fig. S16a). Meanwhile, in the all-carbon analogue, there is an additional unpaired electron originating from the central part (Fig. S16b). The extra electron provided by the nitrogen atom in the N-substituted molecule quenches the central radical, which explains the absence of the central unpaired electron.

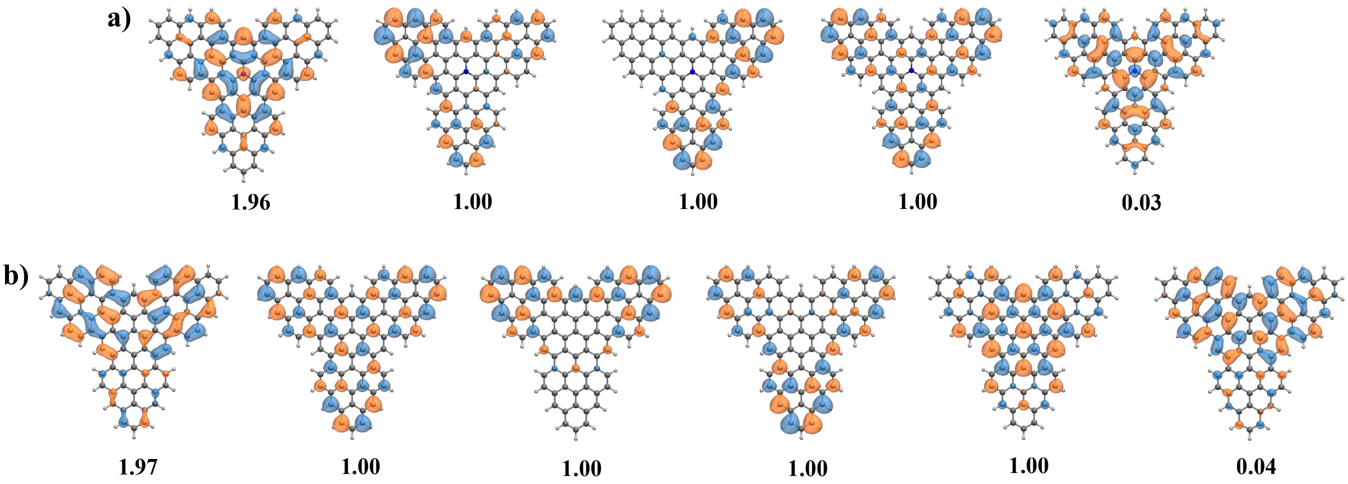


Figure S16: Many-body natural orbitals (with the occupation indicated below), as obtained from the CAS(11,11) calculations for the (a) aza-triangulene molecule and (b) from CAS(12,12) calculations for the fully carbon analogue.

In the purely carbon molecule, the ground state is a quintet ($S = 2$), and the following excited states are two degenerate triplets with an energy gap of 242 meV from the quintet ground state. Fig. S17 shows the dominant Natural Transition Orbitals of spin excitations from the ground state to the lowest excited states. The spin excitation from the quintet to the degenerate triplet states involves only the spins on the edges. Notably, it does not involve the central spin, further indicating that the exchange coupling between the edge spins differs from the coupling between the central spin and the edge spins. The following two excited states are open-shell singlets, which are degenerate and positioned 590 meV higher in energy than the quintet ground state. These states involve spin flips of the unpaired electron at the central part of the carbon-based triangulene.

The exchange coupling (J) between the spins on the external triangulenes in the pure carbon molecule is symmetric, as evidenced by the fact that the first excited state is a doubly degenerate triplet. However, the coupling strength is an order of magnitude higher than that of the N-substituted molecule. Additionally, in the all-carbon molecule, the exchange coupling between the edge spins and the central spin is nearly twice as strong as the coupling between the edge spins, resulting in a double degeneracy in the open-shell singlet.

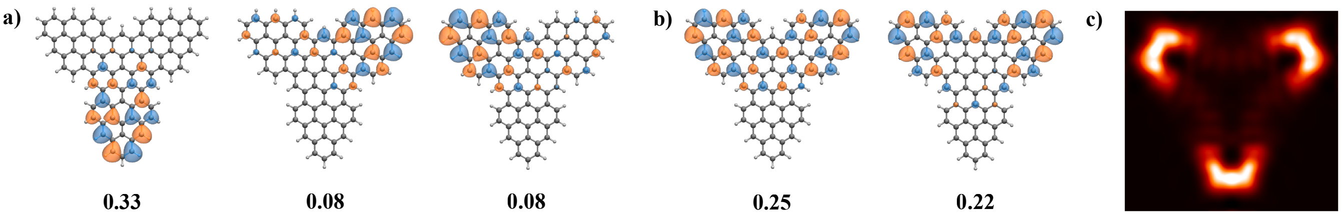


Figure S17: Calculated NTOs from the many-body wavefunctions obtained from CAS(12,12) and their corresponding weights for the spin excitation from the quintet ground state to the degenerate (a) first triplet and (b) second triplet for the fully carbon molecule; c) simulated dI/dV map of NTOs of the spin excitation from the quintet ground state to the degenerate triplet excited states.

# References


(S1) Vilas-Varela, M.; Romero-Lara, F.; Vegliante, A.; Calupitan, J. P.; Martínez, A.; Meyer, L.; Uriarte-Amiano, U.; Friedrich, N.; Wang, D.; Schulz, F.; Koval, N. E.; Sandoval-Salinas, M. E.; Casanova, D.; Corso, M.; Artacho, E.; Peña, D.; Pascual, J. I. On-Surface Synthesis and Characterization of a High-Spin Aza-[5]-Triangulene. *Angew. Chem. Int. Ed.* **2023**, *62*, e202307884.

(S2) Mishra, S.; Yao, X.; Chen, Q.; Eimre, K.; Gröning, O.; Ortiz, R.; Di Giovannantonio, M.; Sancho-García, J. C.; Fernández-Rossier, J.; Pignedoli, C. A.; Müllen, K.; Ruffieux, P.; Narita, A.; Fasel, R. Large Magnetic Exchange Coupling in Rhombus-Shaped Nanographenes with Zigzag Periphery. *Nat. Chem.* **2021**, *13*, 581–586.

(S3) Gross, L.; Mohn, F.; Liljeroth, P.; Repp, J.; Giessibl, F. J.; Meyer, G. Measuring the Charge State of an Adatom with Noncontact Atomic Force Microscopy. *Science* **2009**, *324*, 1428–1431.

(S4) Ishii, H.; Sugiyama, K.; Ito, E.; Seki, K. Energy Level Alignment and Interfacial Electronic Structures at Organic/Metal and Organic/Organic Interfaces. *Adv. Mater.* **1999**, *11*, 605–625.

(S5) Trishin, S.; Müller, T.; Rolf, D.; Lotze, C.; Rietsch, P.; Eigler, S.; Meyer, B.; Franke, K. J. Resolution of Intramolecular Dipoles and a Push-Back Effect of Individual Molecules on a Metal Surface. *J. Phys. Chem. C* **2022**, *126*, 7667–7673.

(S6) Leoni, T.; Guillermet, O.; Walch, H.; Langlais, V.; Scheuermann, A.; Bonvoisin, J.; Gauthier, S. Controlling the Charge State of a Single Redox Molecular Switch. *Phys. Rev. Lett.* **2011**, *106*, 216103.

(S7) Gao, Y.; Albrecht, F.; Rončević, I.; Ettedgui, I.; Kumar, P.; Scriven, L. M.; Christensen, K. E.; Mishra, S.; Righetti, L.; Rossmannek, M.; Tavernelli, I.; Anderson, H. L.; Gross, L. On-surface synthesis of a doubly anti-aromatic carbon allotrope. *Nature* **2023**, *623*, 977–981.

(S8) Li, J.; Sanz, S.; Corso, M.; Choi, D. J.; Peña, D.; Frederiksen, T.; Pascual, J. I. Single spin localization and manipulation in graphene open-shell nanostructures. *Nat. Commun.* **2019**, *10*, 200.

(S9) Li, J.; Sanz, S.; Castro-Esteban, J.; Vilas-Varela, M.; Friedrich, N.; Frederiksen, T.; Peña, D.; Pascual, J. I. Uncovering the Triplet Ground State of Triangular Graphene Nanoflakes Engineered with Atomic Precision on a Metal Surface. *Phys. Rev. Lett.* **2020**, *124*, 177201.

(S10) Zhang, Y.-h.; Kahle, S.; Herden, T.; Stroh, C.; Mayor, M.; Schlickum, U.; Ternes, M.; Wahl, P.; Kern, K. Temperature and magnetic field dependence of a Kondo system in the weak coupling regime. *Nat. Commun.* **2013**, *4*, 2110.

(S11) Neese, F. The ORCA Program System. *WIREs Comput. Mol. Sci.* **2012**, *2*, 73–78.

(S12) Blum, V.; Gehrke, R.; Hanke, F.; Havu, P.; Havu, V.; Ren, X.; Reuter, K.; Scheffler, M. Ab Initio Molecular Simulations with Numeric Atom-Centered Orbitals. *Comput. Phys. Commun.* **2009**, *180*, 2175–2196.

(S13) Adamo, C.; Cossi, M.; Barone, V. An accurate density functional method for the study of magnetic properties: the PBE0 model. *J. Mol. Struct.: THEOCHEM* **1999**, *493*, 145–157.

(S14) Martin, R. L. Natural Transition Orbitals. *J. Chem. Phys.* **2003**, *118*, 4775–4777.

(S15) Calvo-Fernández, A.; Kumar, M.; Soler-Polo, D.; Eiguren, A.; Blanco-Rey, M.; Jelínek, P. Theoretical model for multiorbital Kondo screening in strongly correlated molecules with several unpaired electrons. *Phys. Rev. B* **2024**, *110*, 165113.

(S16) Zuzak, R.; Kumar, M.; Stoica, O.; Soler-Polo, D.; Brabec, J.; Pernal, K.; Veis, L.; Blieck, R.; Echavarren, A. M.; Jelinek, P.; Godlewski, S. On-Surface Synthesis and Determination of the Open-Shell Singlet Ground State of Tridecacene**. *Angew. Chem. Int. Ed.* **2024**, *63*, e202317091.

(S17) Ortiz, J. V. Dyson-Orbital Concepts for Description of Electrons in Molecules. *J. Chem. Phys.* **2020**, *153*, 070902.

(S18) Krejčí, O.; Hapala, P.; Ondráček, M.; Jelínek, P. Principles and simulations of high-resolution STM imaging with a flexible tip apex. *Phys. Rev. B* **2017**, *95*, 045407
.
